# Development and evaluation of a transesophageal echocardiography–derived left atrial appendage structure–function coupling index across the atrial fibrillation disease continuum

**DOI:** 10.3389/fcvm.2026.1951089

**Published:** 2026-10-28

**Authors:** Jianbo Zhu, Chen Ni, Yangnan Ruan, Yanfeng Jiang, Lei Zhu, Yanhua Huang, Zhenzhen Jiang, Xiatian Liu

**Affiliations:** 1 Department of Echocardiography, Shaoxing People’s Hospital, Shaoxing, China; 2 Department of Echocardiography, The First Affiliated Hospital, Shaoxing University, Shaoxing, China; 3 School of Medicine, Shaoxing University, Shaoxing, China

**Keywords:** atrial cardiomyopathy, atrial fibrillation, coupling index, global longitudinal strain, left atrial appendage, speckle-tracking echocardiography, stroke, transesophageal echocardiography

## Abstract

**Background:**

Atrial fibrillation (AF) is a progressive disorder associated with atrial remodeling and thromboembolic risk. We developed a transesophageal echocardiography (TEE)-derived left atrial appendage (LAA) structure-function coupling index (LAA-SFCI), defined as LAA volumetric load relative to longitudinal deformation, and assessed its distribution along the AF disease continuum and its potential for risk re-stratification of patients without documented AF.

**Methods:**

This single-center, prospective, cross-sectional study included 267 patients undergoing clinically indicated TEE, classified by the 2023 American College of Cardiology/American Heart Association/American College of Clinical Pharmacy/Heart Rhythm Society (ACC/AHA/ACCP/HRS) framework as Stage 1 (*n* = 68), Stage 2 (*n* = 65), Stage 3a (*n* = 66), or Stage 3b (*n* = 68). Seven candidate LAA-SFCIs were calculated as the LAA volume index (LAA-VI) divided by a functional measure, and the principal index was selected by ordinal logistic regression using the Akaike information criterion. Discrimination was compared using the DeLong test and internally validated by bootstrapping, and patients without documented AF (Stages 1–2) were stratified by the exploratory cutoff.

**Results:**

LAA-VI/LAA-GLS (LAA global longitudinal strain) provided the best model fit and rose across AF stages from 0.33 ± 0.11 to 0.86 ± 0.36 (mL/m^2^)/% (*P* < 0.001). In ordinal regression with the log-transformed index, each 1-SD increase remained associated with higher AF stage after adjustment for age, sex, body mass index, left ventricular ejection fraction, and left atrial volume index (odds ratio 11.04, 95% confidence interval 7.17–16.98). For Stage 1–2 versus Stage 3a–3b, its area under the curve (0.904; optimism-corrected 0.900) was significantly higher than those of LAA-VI (0.661) and LAA-GLS (0.824) (both DeLong *P* < 0.001). Among 133 patients without documented AF, 18 (13.5%) exceeded the cutoff of 0.460 (mL/m^2^)/%; these patients did not differ from the others in left atrial volume index or age.

**Conclusion:**

In this cross-sectional study, LAA-VI/LAA-GLS characterized LAA structure–function uncoupling along the AF disease continuum. In patients without documented AF undergoing clinically indicated TEE, it identified a subgroup with an AF-like LAA phenotype not captured by conventional atrial size measures. These findings are hypothesis-generating and require external validation and longitudinal follow-up to determine their prognostic value.

## 1 Introduction

Atrial fibrillation (AF) is associated with ischemic stroke, heart failure, cognitive decline, and increased mortality (1–3) and is increasingly recognized as a progressive disease that develops along a continuum from risk-factor accumulation and atrial remodeling to clinical arrhythmia (4, 5). The 2023 ACC/AHA/ACCP/HRS guideline formalized this concept as the AF disease continuum: Stage 1 (at risk) denotes the presence of modifiable or non-modifiable AF risk factors; Stage 2 (pre-AF) denotes structural or electrical atrial abnormalities without documented AF; Stage 3 denotes clinical AF, including paroxysmal (3a) and persistent (3b) AF; and Stage 4 denotes permanent AF (2). By shifting attention upstream to the at-risk and pre-AF stages, this framework emphasizes that atrial remodeling may be present before AF is first documented.

The left atrial appendage (LAA) is a key anatomical target of AF-related atrial cardiomyopathy and local thrombus formation (6). Through active contraction, the LAA promotes emptying and limits local blood stasis (7, 8); as atrial remodeling advances, LAA dilatation, reduced deformation, and slower flow favor spontaneous echo contrast (SEC) and thrombus formation (9), and LAA structural and functional abnormalities may therefore represent a local manifestation of atrial cardiomyopathy (10). Transesophageal echocardiography (TEE) provides close-range visualization of LAA anatomy, flow, SEC, and thrombus (9, 11), and two-dimensional speckle-tracking echocardiography (2D-STE) applied to TEE images quantifies LAA global longitudinal strain (LAA-GLS), which reflects myocardial deformation rather than intracavitary flow (12–15).

However, current tools capture local LAA remodeling only indirectly. The CHA₂DS₂-VASc score integrates systemic clinical risk factors but does not characterize the LAA itself, although more than 90% of thrombi in nonvalvular AF originate in the LAA (16). Among imaging measures, the left atrial volume index (LAVI) and the LAA volume index (LAA-VI) quantify chamber enlargement but not myocardial deformation (17); LAA emptying velocity is strongly influenced by rhythm, heart rate, and loading conditions at examination (18); and LAA-GLS reflects deformation without conveying the volumetric burden against which that deformation occurs (15). Consequently, patients without documented AF who share similar clinical risk profiles and atrial size may differ substantially in the extent of local LAA remodeling, and this heterogeneity cannot be resolved by any single structural or functional parameter.

Structure–function coupling indices address this limitation by relating the load imposed on a cardiac chamber to its mechanical performance, as exemplified by ventricular–arterial coupling, right ventricular–pulmonary arterial coupling, and the left atrial stiffness index, each of which provides information beyond its individual components (19–21).

Against this background, we developed a TEE-based LAA structure–function coupling index (LAA-SFCI) framework in which LAA volumetric burden was paired with different LAA functional measures to form a family of candidate indices. Using the 2023 AF stages as a reference continuum, we hypothesized that LAA structure–function uncoupling would be greater at higher AF stages. We aimed to (1) construct candidate LAA-SFCIs and identify the principal index; (2) characterize its distribution across Stages 1, 2, 3a, and 3b and compare its discrimination with that of its individual components and LAVI; and (3) explore whether, among patients without documented AF who were already undergoing clinically indicated TEE, the index could identify a subgroup whose LAA phenotype resembled that of clinical AF, as a basis for risk re-stratification.

## 2 Materials and methods

### 2.1 Study population and design

This was a single-center, prospective, cross-sectional observational study, with analysis performed according to predefined AF disease stages. Adult patients who underwent clinically indicated TEE at our center between March 2024 and March 2026 were consecutively screened to constitute the source population. This source population comprised patients with nonvalvular AF, as well as patients without a documented history of clinical AF who underwent TEE for other clinical indications, including patent foramen ovale (PFO) screening and evaluation of cryptogenic stroke or a cardioembolic source. Patients without a history of clinical AF served only as a potential source population for AF Stage 1 or Stage 2, not as healthy controls. Among patients without clinical AF (Stages 1 and 2), all TEE examinations were requested by the treating physicians to evaluate clinically suspected PFO, on the basis of either a prior stroke, transient ischemic attack (TIA), or thromboembolism (Stage 1, *n* = 12 [17.6%]; Stage 2, *n* = 12 [18.5%]) or migraine (Stage 1, *n* = 56 [82.4%]; Stage 2, *n* = 53 [81.5%]), as ascertained from the medical records; PFO was confirmed by TEE in 17 (25.0%) and 11 (16.9%) patients, respectively (Figure 1 and Table 1); among patients with clinical AF (Stages 3a and 3b), TEE was performed mainly to assess for left atrial or LAA thrombus. No TEE was performed solely for research purposes. Indication-related characteristics, including prior stroke, TIA, or thromboembolism, migraine, and the presence of PFO, are reported by stage in Table 1.

**Figure 1:**
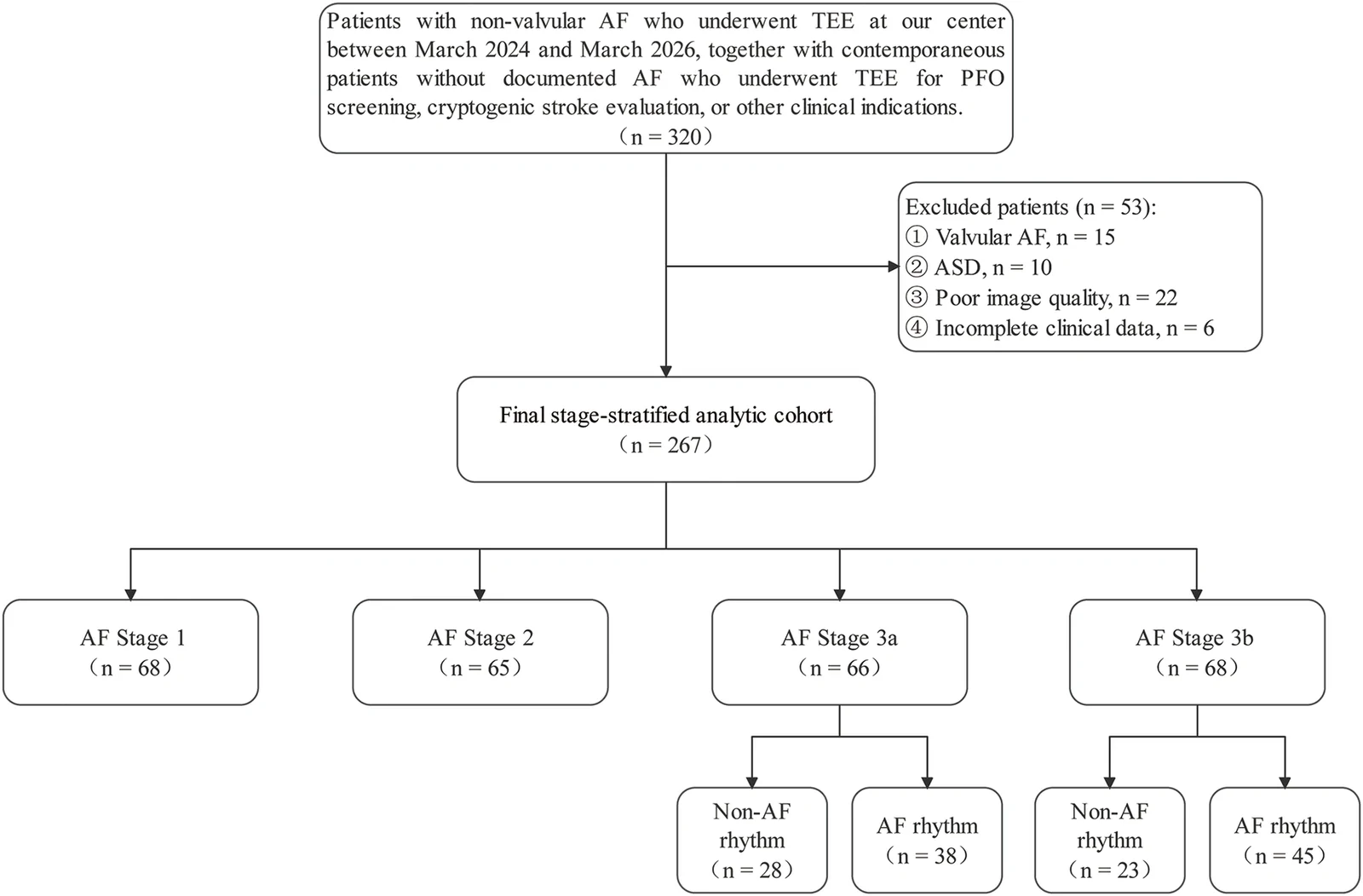
Study flowchart and AF disease-stage classification. AF, atrial fibrillation; ASD, atrial septal defect; PFO, patent foramen ovale; TEE, transesophageal echocardiography.

**Table 1:** Baseline clinical characteristics across AF disease stages.

Variables	AF Stage 1 (*n* = 68)	AF Stage 2 (*n* = 65)	AF Stage 3a (*n* = 66)	AF Stage 3b (*n* = 68)	*P-* value
Age, y, Mean ± SD	65.66 ± 8.62	62.23 ± 9.94	65.52 ± 8.95	66.82 ± 8.31	0.024
Female, *n* (%)	20 (29.41)	28 (43.08)	24 (36.36)	26 (38.24)	0.429
Body mass index, kg/m^2^	24.49 ± 4.10	25.01 ± 3.55	25.51 ± 5.19	24.72 ± 4.23	0.555
BSA, m^2^, Mean ± SD	1.57 ± 0.14	1.58 ± 0.12	1.60 ± 0.16	1.57 ± 0.14	0.701
Carotid arteriosclerosis, *n* (%)	35 (51.47)	28 (43.08)	36 (54.55)	36 (52.94)	0.562
Coronary heart disease, *n* (%)	4 (5.88)	11 (16.92)	11 (16.67)	6 (8.82)	0.116
Diabetes mellitus, *n* (%)	8 (11.76)	13 (20.00)	6 (9.09)	9 (13.24)	0.316
Hypertension, *n* (%)	31 (45.59)	33 (50.77)	42 (63.64)	43 (63.24)	0.082
Alcohol history, *n* (%)	22 (32.35)	20 (30.77)	17 (25.76)	19 (27.94)	0.841
Smoking history, *n* (%)	24 (35.29)	15 (23.08)	22 (33.33)	16 (23.53)	0.275
Stroke, *n* (%)	0 (0.00)	2 (3.08)	7 (10.61)	16 (23.53)	<0.001
Prior stroke, TIA, or thromboembolism, *n* (%)	12 (17.65)	12 (18.46)	12 (18.18)	25 (36.76)	0.018
Migraine, *n* (%)	56 (82.35)	53 (81.54)	0 (0.00)	0 (0.00)	<0.001
PFO, *n* (%)	17 (25.00)	11 (16.92)	15 (22.73)	10 (14.71)	0.398
AF rhythm during TEE, *n* (%)	0 (0.00)	0 (0.00)	38 (57.58)	45 (66.18)	<0.001
SEC, *n* (%)					<0.001
Grade 0	68 (100.00)	10 (15.38)	0 (0.00)	0 (0.00)	
Grade 1	0 (0.00)	55 (84.62)	48 (72.73)	0 (0.00)	
Grade 2	0 (0.00)	0 (0.00)	18 (27.27)	23 (33.82)	
Grade 3	0 (0.00)	0 (0.00)	0 (0.00)	36 (52.94)	
Grade 4	0 (0.00)	0 (0.00)	0 (0.00)	9 (13.24)	
LAA thrombus, *n* (%)	0 (0.00)	0 (0.00)	2 (3.03)	5 (7.35)	0.016
CHA_2_DS_2_-VASc score, Mean ± SD	1.74 ± 1.03	2.17 ± 1.24	2.35 ± 1.44	2.47 ± 1.11	0.003

Values are presented as mean ± SD or *n* (%). LAA thrombus was compared with the Fisher–Freeman–Halton exact test. Migraine was compared with the Fisher–Freeman–Halton exact test. AF, atrial fibrillation; BSA, body surface area; CHA₂DS₂-VASc, congestive heart failure, hypertension, age ≥75 years, diabetes mellitus, stroke/transient ischemic attack/thromboembolism, vascular disease, age 65–74 years, and sex category; LAA, left atrial appendage; PFO, patent foramen ovale; SEC, spontaneous echo contrast; TEE, transesophageal echocardiography; TIA, transient ischemic attack; SD, standard deviation.

All eligible patients underwent standardized transthoracic echocardiography (TTE) and TEE. AF disease stage was determined by integrating clinical history, prior arrhythmia records, 12-lead electrocardiography, Holter monitoring, and conventional TTE data from the hospitalization or examination cycle in which the index TEE was performed. The staging definitions and eligibility criteria are detailed in Section 2.2. The interval between TTE and TEE was limited to no more than 7 days, and no electrical or pharmacological cardioversion, catheter ablation, or other clinical event likely to substantially alter atrial function or hemodynamic status occurred between the two examinations. The actual cardiac rhythm and heart rate at the time of TEE were recorded. Importantly, to avoid circularity, AF stage was assigned exclusively based on clinical history, 12-lead electrocardiography, ambulatory Holter monitoring, and TTE; LAVI was measured but was not used as a staging criterion (Section 2.2); no TEE-derived measurement, LAA structural or functional parameter, or SEC grade was used at any point in stage assignment. AF stage was independently adjudicated by two cardiologists blinded to all TEE-derived LAA data and SEC grades, with disagreements resolved by a third senior cardiologist. All TEE-derived LAA parameters and SEC grades were subsequently measured and graded by observers blinded to clinical stage, ensuring that stage assignment and the index under study were determined independently.

The sample size was estimated *a priori* using one-way analysis of variance (ANOVA) across the four groups, with the between-group difference in LAA structure–function parameters across the four AF stages as the primary endpoint. Because the optimal structure–function coupling index had not been determined at the design stage, the sample size could not be based on the index itself. Instead, a conservative, moderate effect size that is independent of the observed data and widely accepted as a benchmark (Cohen's f = 0.25) was assumed *a priori* to avoid overestimating the effect and thereby underestimating the required sample size. Using G*Power 3.1, with a two-sided *α* of 0.05, a power of 0.90, and equal allocation across the four groups, at least 58 analyzable cases per group were required.

Accordingly, a stratified quota consecutive enrollment strategy was applied, with an enrollment cap of 80 patients per group, to achieve statistical power while maintaining balanced sample sizes across stages. Throughout the enrollment window, all eligible patients were screened consecutively in order of presentation and assigned to AF Stage 1, 2, 3a, or 3b according to the criteria in Section 2.2. Each stage group was counted independently; once a group reached the cap of 80, enrollment into that group was stopped, and enrollment continued only for groups that had not yet reached the cap, until all four groups reached 80. Before a group was full, eligible patients were enrolled consecutively and without selection in order of presentation, thereby minimizing selection bias. After 80 patients had been enrolled in each of the four groups (320 in total), the inclusion and exclusion criteria were applied, excluding 53 patients (16.6%) because of valvular AF (*n* = 15), atrial septal defect (*n* = 10), inadequate TEE image quality (*n* = 22), or incomplete clinical data (*n* = 6). This left 68, 65, 66, and 68 patients for analysis in Stage 1, 2, 3a, and 3b, respectively (267 in total), each exceeding the required threshold of 58 per group for a medium effect (Cohen's f = 0.25) (Figure 1). Details of the sample-size estimation are provided in Supplementary Material S1 and Supplementary Tables S1, S2.

### 2.2 Clinical staging and eligibility criteria

According to the AF disease-continuum concept proposed in the 2023 ACC/AHA/ACCP/HRS AF guideline and predefined staging criteria, patients were classified into four groups: AF Stage 1, Stage 2, Stage 3a, and Stage 3b. Stage 1 was defined as the at-risk stage, with no prior evidence of clinical AF but one or more AF-related risk factors. AF-related risk factors comprised advanced age (≥65 years), hypertension, diabetes mellitus, coronary heart disease, carotid atherosclerosis, obesity (body mass index ≥28 kg/m^2^), smoking, alcohol consumption, and prior stroke, TIA, or thromboembolism. Stage 2 (pre-AF) was defined as no documented clinical AF together with evidence of atrial electrical abnormality on 24-h ambulatory Holter monitoring, defined as frequent atrial premature complexes (≥500/24 h) or non-sustained atrial tachycardia (≥3 consecutive atrial beats at >100 bpm lasting <30 s). Although left atrial enlargement is also recognized as a pre-AF marker in the 2023 guideline, LAVI was not used as a staging criterion in this study; consequently, some patients classified as Stage 1 had a study-measured LAVI > 34 mL/m^2^ (Table 2). A sensitivity analysis reclassifying these patients as Stage 2 is provided in Supplementary Table S11. Stage 3a was defined as paroxysmal AF, in which episodes terminate spontaneously within 7 days. Stage 3b was defined as persistent AF, in which AF lasts more than 7 days, requires pharmacological or electrical cardioversion for termination, or is explicitly documented as persistent AF in the medical record.

**Table 2:** Conventional TTE parameters and TEE-derived LAA structural and functional parameters across AF disease stages.

Variables	AF Stage 1 (*n* = 68)	AF Stage 2 (*n* = 65)	AF Stage 3a (*n* = 66)	AF Stage 3b (*n* = 68)	*P-* value
TTE Parameters					
LAD, mm	38.56 ± 5.85	40.23 ± 6.45	41.76 ± 5.63	41.88 ± 6.47	0.005
LAVI, mL/m^2^	36.69 ± 3.41	38.99 ± 4.54	38.74 ± 3.60	41.31 ± 5.90	<0.001
LVEF, %	58.13 ± 4.99	56.62 ± 11.42	47.44 ± 9.30	47.06 ± 9.67	<0.001
TEE-derived LAA structural parameters					
Maximum LAA ostial area, cm^2^	2.74 ± 0.61	3.36 ± 1.38	3.48 ± 1.16	3.43 ± 1.16	<0.001
Maximum LAA depth, mm	28.71 ± 0.71	29.00 ± 0.91	28.84 ± 0.87	28.86 ± 0.82	0.239
LAA-EDV, mL	7.33 ± 1.86	7.98 ± 1.72	8.23 ± 1.70	9.80 ± 2.31	<0.001
LAA-ESV, mL	4.60 ± 1.33	5.33 ± 1.18	5.82 ± 1.22	7.45 ± 1.28	<0.001
LAA-VI, mL/m^2^	4.73 ± 1.34	5.08 ± 1.18	5.24 ± 1.34	6.28 ± 1.63	<0.001
TEE-derived LAA functional parameters					
LAA-PEV, cm/s	53.14 ± 12.72	53.66 ± 17.45	48.30 ± 14.47	39.52 ± 17.67	<0.001
LAA-PFV, cm/s	53.22 ± 17.25	57.93 ± 17.98	50.90 ± 15.31	46.41 ± 18.22	0.002
LAA-EF, %	36.42 ± 13.06	31.03 ± 17.17	28.62 ± 13.35	21.89 ± 12.92	<0.001
LAA-SLE, mm	19.60 ± 4.15	15.94 ± 4.05	12.43 ± 4.05	9.17 ± 3.96	<0.001
LAA-s′, cm/s	28.32 ± 7.22	23.17 ± 10.32	23.20 ± 13.43	21.54 ± 9.62	<0.001
LAA-FAC, %	28.30 ± 2.89	26.11 ± 4.01	30.28 ± 5.35	20.71 ± 3.53	<0.001
LAA-GLS, %	14.90 ± 3.84	12.69 ± 3.39	9.65 ± 2.98	8.37 ± 3.48	<0.001

Values are presented as mean ± SD. AF, atrial fibrillation; EDV, end-diastolic volume; ESV, end-systolic volume; FAC, fractional area change; GLS, global longitudinal strain; LAA, left atrial appendage; LAA-EF, left atrial appendage emptying fraction; LAA-SLE, left atrial appendage systolic longitudinal excursion; LAD, left atrial diameter; LAVI, left atrial volume index; LVEF, left ventricular ejection fraction; PFV, peak filling velocity; PEV, peak emptying velocity; LAA-s′, left atrial appendage peak systolic tissue velocity; SD, standard deviation; TEE, transesophageal echocardiography; TTE, transthoracic echocardiography; VI, volume index.

The AF continuum also includes Stage 3c, Stage 3d, and Stage 4, none of which was included in the primary analysis cohort. Stage 3c represents long-standing persistent AF and was not included because the number of eligible cases meeting all study criteria at our center was limited. Stage 3d represents the post-treatment state of maintained freedom from AF after successful catheter or surgical ablation, in which LAA structure and function may be affected by ablation and rhythm restoration. Stage 4 represents permanent AF, in which the patient and clinician have jointly decided to forgo further rhythm-control attempts, and is characterized by substantial heterogeneity in AF duration, prior treatment strategy, and the degree of atrial remodeling.

The inclusion criteria were as follows: (1) age ≥18 years; (2) completion of TTE and TEE for clinical indications such as nonvalvular AF, PFO screening, cryptogenic stroke, or evaluation of a cardioembolic source; (3) complete clinical, electrocardiographic, or Holter data required for AF staging (Stage 1 to Stage 3b); and (4) echocardiographic images suitable for analysis of LAA structure, function, and 2D-STE.

The exclusion criteria were as follows: (1) valvular AF; (2) a history of cardiac surgery, pacemaker implantation, or LAA intervention; (3) cardiac tumor; (4) congenital heart disease other than isolated PFO; and (5) inadequate TEE image quality precluding analysis of LAA structural, functional, or 2D-STE parameters.

### 2.3 Echocardiographic acquisition and offline analysis

Echocardiographic examinations were performed using a GE Vivid E95 Ultra Edition ultrasound system (GE Vingmed Ultrasound, Horten, Norway) equipped with a transthoracic phased array probe M5Sc-D (1.4–4.6 MHz) and a 4D matrix array multiplane transesophageal probe 6VT-D (3–8 MHz). All images were acquired and stored according to a standardized institutional protocol. Offline measurements, three-dimensional image analysis, volumetric assessment, and 2D-STE-derived strain analysis were performed with EchoPAC PC software, version 204 (GE Vingmed Ultrasound, Horten, Norway). LAA structural, flow, motion, volumetric, and strain parameters were measured offline by investigators blinded to clinical group assignment. In patients in sinus rhythm, measurements were averaged over 3 consecutive cardiac cycles. In patients in AF rhythm, measurements were averaged over 5 consecutive cardiac cycles with adequate image quality to minimize the influence of beat-to-beat variability; the index-beat method was not applied.

### 2.4 Clinical and thrombus-related variables

All clinical data were extracted by two uniformly trained investigators using a standardized data-collection form and were systematically cross-checked against the hospital's integrated electronic medical record system, the echocardiography reporting system, and the electrophysiology laboratory database; the collected clinical variables are listed in Table 1. BSA was calculated using the Mosteller formula: BSA (m^2^) = √ [height (cm) × weight (kg)/3600]. The CHA₂DS₂-VASc score was calculated from heart failure, hypertension, age, diabetes mellitus, prior stroke or transient ischemic attack, vascular disease, and sex category. Rhythm status during TEE was determined from the simultaneous electrocardiographic recording and categorized as sinus rhythm or AF rhythm. SEC was defined as dynamic smoke-like echogenicity within the left atrium or LAA on TEE and was graded from 0 to 4 according to echo intensity, following Fatkin et al. (22): grade 0, no SEC; grade 1, minimal SEC detectable only after gain augmentation or transiently during part of the cardiac cycle; grade 2, mild SEC visible under standard gain settings; grade 3, moderate-to-severe SEC presenting as persistent, prominent swirling smoke-like echoes throughout the cardiac cycle; and grade 4, severe SEC with intense, dense echogenicity and very slow, sluggish swirling within the LAA. LAA thrombus was defined as an abnormal echogenic mass within the LAA cavity that was clearly distinguishable from the endocardium and pectinate muscles (9, 11). SEC grading and LAA thrombus assessment were performed independently by two physicians experienced in TEE; disagreements were resolved by consensus after review by a third senior physician.

#### 2.4.1 Conventional TTE parameter measurements

Conventional TTE parameters included left atrial anteroposterior diameter (LAD), left atrial volume index (LAVI), and left ventricular ejection fraction (LVEF). LAD was measured in the parasternal left ventricular long-axis view at left ventricular end-systole. Left atrial volume and LVEF were derived from two separately acquired sets of apical views rather than from a single biplane acquisition. Left atrial volume was measured from LA-focused apical four- and two-chamber views: during acquisition, the probe orientation was adjusted to display the left atrium fully and to avoid left atrial foreshortening, so that the left ventricle often appeared foreshortened in these views. Maximal left atrial volume (LAVmax) was obtained at left ventricular end-systole, immediately before mitral valve opening, using the modified Simpson method, and was indexed to BSA to yield LAVI: LAVI=LAVmax/BSA. LVEF was measured with the modified Simpson method from standard LV-focused apical four- and two-chamber views at left ventricular end-diastole and end-systole; these views were optimized for the left ventricle and had imaging axes different from those of the LA-focused views. All measurements were performed in accordance with the American Society of Echocardiography/European Association of Cardiovascular Imaging (ASE/EACVI) chamber quantification guidelines (23).

#### 2.4.2 TEE-derived LAA structural parameter measurement

TEE-derived LAA structural parameters included maximum LAA ostial area, maximum LAA depth, LAA end-diastolic volume (LAA-EDV), LAA end-systolic volume (LAA-ESV) and LAA-VI. All LAA structural parameters were measured offline by investigators blinded to clinical group assignment, as follows.

(1) Maximum LAA ostial area. Maximum LAA ostial area was measured from three-dimensional TEE volumetric datasets. After a complete three-dimensional full-volume dynamic dataset of the LAA had been acquired, the data were imported into EchoPAC PC software, for offline analysis. Using multiplanar reconstruction, the image plane was adjusted to be as perpendicular as possible to the LAA long axis while fully displaying the LAA ostium boundary. When the ostium was most clearly visualized and reached its maximal area, its inner border was manually traced to obtain the maximum LAA ostial area.
(2) Maximum LAA depth. Maximum LAA depth was defined as the maximum distance from the center of the LAA ostial plane to the most distal LAA tip, measured along the main long axis of the LAA.
(3) LAA volumetric parameters. LAA volume was assessed using a biplane method. Cardiac cycles with complete LAA visualization, clear endocardial borders, and no obvious artifacts were selected. Two orthogonal biplane view pairs, 0°/90° and 45°/135°, were used to display the LAA long axis and its perpendicular plane, and the LAA endocardial border was traced manually or semi-automatically, with adjustments made according to the actual contour of the LAA cavity while excluding the left atrial body, the pulmonary vein ostia, and surrounding non-LAA structures. LAA-EDV was defined as the LAA volume at maximal filling during a cardiac cycle, and LAA-ESV as the minimal cavity volume after LAA emptying. The mean values from the two sets of biplane measurements were used as the final LAA-EDV and LAA-ESV for subsequent analyses.
(4) LAA-VI. LAA-VI was defined as LAA-EDV indexed to BSA and calculated as follows: LAA-VI = LAA-EDV/BSA.

#### 2.4.3 TEE-derived LAA functional parameter measurement

TEE-derived LAA functional parameters included LAA peak emptying velocity (LAA-PEV), LAA peak filling velocity (LAA-PFV), and LAA emptying fraction (LAA-EF), LAA systolic longitudinal excursion (LAA-SLE), LAA peak systolic tissue Doppler velocity (LAA-s′), LAA fractional area change (LAA-FAC), and LAA-GLS, measured as follows.

(1) LAA flow velocity parameters. LAA-PEV and LAA-PFV were measured with spectral Doppler, with the sample volume placed within the proximal-to-mid LAA cavity and aligned as parallel as possible to the direction of blood flow; they represented peak LAA emptying and filling velocities, respectively.
(2) LAA volumetric emptying function. LAA-EF was calculated from LAA-EDV and LAA-ESV to reflect global LAA volumetric emptying function: LAA-EF = (LAA-EDV−LAA-ESV)/LAA-EDV×100%.
(3) LAA longitudinal excursion parameter. LAA-SLE was a study-defined LAA longitudinal motion parameter used to quantify systolic excursion of the LAA wall along its long axis, conceptually analogous to tricuspid and mitral annular plane systolic excursion (TAPSE/MAPSE). It was measured in the TEE 45° view by aligning the M-mode cursor along the LAA long axis, from the ostium to the most distal tip, and recording the systolic longitudinal excursion of the LAA wall (Figure 2).
(4) LAA tissue Doppler velocity parameter. LAA-s′ was a study-defined LAA motion parameter, conceptually adapted from the tissue Doppler s′ used in right ventricular functional assessment. All LAA-s′ measurements were performed in the 45° TEE view. In tissue Doppler imaging mode, the sample volume was placed at the most distal LAA tip and aligned as parallel as possible to the direction of LAA long-axis motion. LAA-s′ was defined as the longitudinal traveling velocity in this region during the LAA emptying/contraction-related phase and was used to reflect local LAA myocardial motion capacity (Figure 2).
(5) LAA area-change parameter. LAA-FAC was a study-defined LAA area-change parameter, conceptually adapted from the fractional area change used in right ventricular functional assessment. All LAA-FAC measurements were performed in the 45° TEE view. In the same TEE view, the LAA endocardial border was manually traced at the maximal filling and minimal emptying phases to obtain the maximal and minimal LAA areas. LAA-FAC was calculated as follows: LAA-FAC=(maximal LAA area−minimal LAA area)/maximal LAA area×100%.
(6) LAA strain parameter. LAA-GLS was derived from STE analysis and defined as the average peak longitudinal strain of the analyzable LAA segments, reflecting global longitudinal mechanical deformation of the LAA. Two-dimensional TEE cine images with complete LAA visualization, clear endocardial borders, a frame rate of 50–60 frames/s (sector width and imaging depth were narrowed to maximize frame rate), and no obvious artifacts or dropout were selected. Following previous LAA-STE approaches, strain analysis was performed in four mid-esophageal TEE views (0°, 45°, 90°, and 135°). Because dedicated LAA strain software is not available, the conventional two-dimensional strain tool of EchoPAC developed for the left ventricle was used, as in previous TEE studies (14). In all patients, regardless of rhythm, the R wave of the simultaneous ECG was used as the zero-strain reference, and each analyzed cardiac cycle was defined from one R wave to the next (R–R gating); P-wave gating was not used because discrete P waves are absent during AF (11, 13). In EchoPAC, the LAA endocardial border was manually traced in each view, and the automatically generated region of interest was adjusted as needed to cover the full thickness of the LAA wall while avoiding inclusion of the left atrial body, the pulmonary vein ostia, intracavitary flow signals, regions of excessive pectinate-muscle interference, and surrounding non-LAA tissue. After automated tracking of LAA wall motion and generation of segmental longitudinal strain curves, tracking quality was inspected segment by segment; segments with suboptimal tracking were readjusted and reanalyzed, and those for which reliable tracking still could not be achieved were excluded from the final calculation. Patients in whom LAA structural, functional, or STE parameters could not be reliably analyzed because of inadequate TEE image quality were excluded (*n* = 22; Figure 1); consequently, LAA-GLS was available in all 267 patients in the final cohort. LAA-GLS for each view was defined as the average peak longitudinal strain of all analyzable LAA segments in that view (the global strain value automatically reported by the software for each cycle, averaged over 3 consecutive cycles in sinus rhythm and 5 consecutive cycles in AF without index-beat selection), and the final LAA-GLS was calculated as the mean of the values from the 0°, 45°, 90°, and 135° views. Because EchoPAC exports longitudinal strain as signed values, the absolute magnitude of LAA-GLS was used to represent LAA longitudinal mechanical deformation capacity; when the software output was negative, its absolute value was used for statistical analysis and LAA-SFCI calculation. LAA-GLS is therefore reported as an absolute value throughout this study (Figure 2).

**Figure 2:**
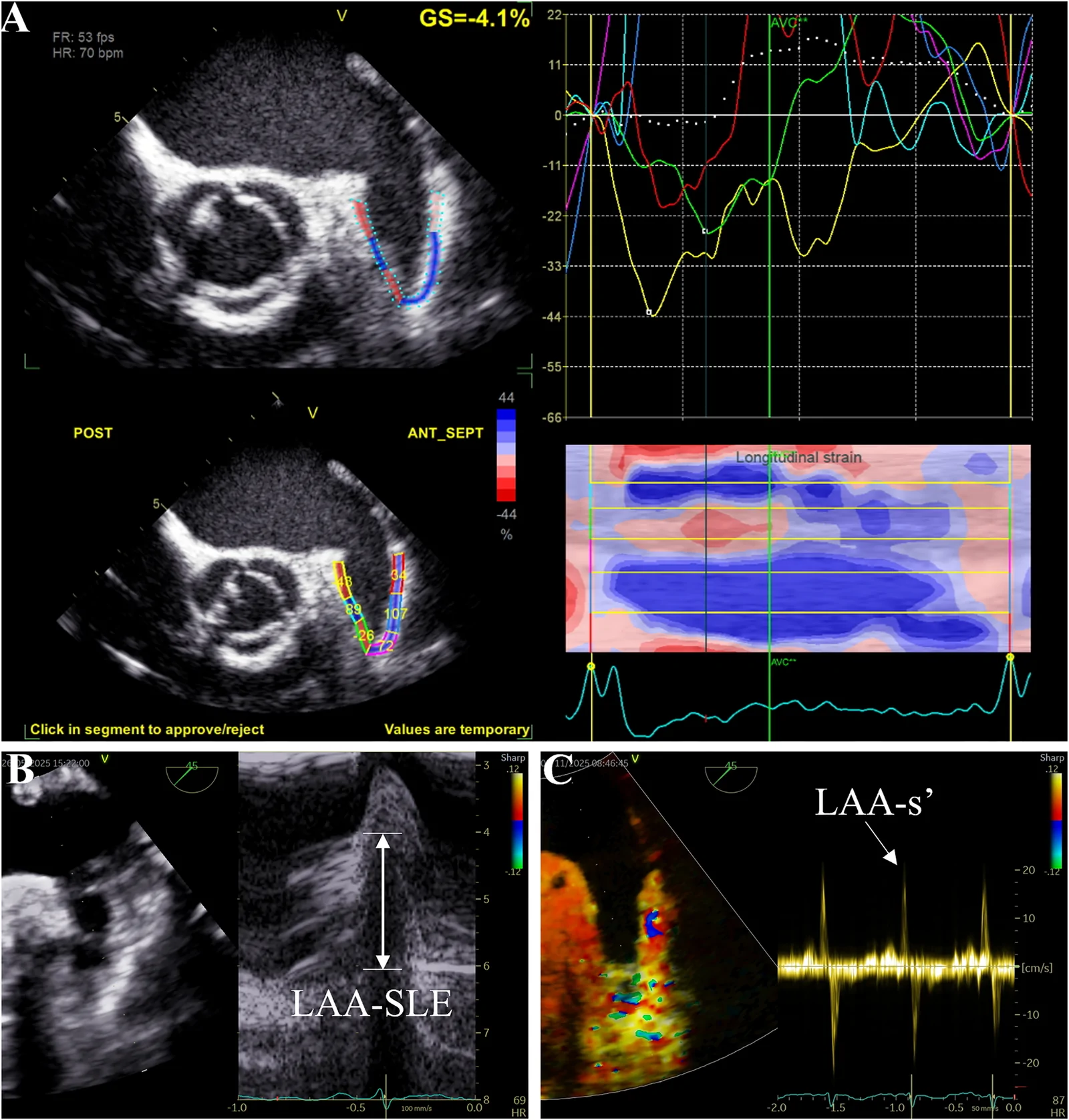
Representative TEE images of LAA mechanical function assessment. **(A)** LAA-GLS analysis. The LAA endocardial border was manually traced by 2D-STE, and the ROI was adjusted to cover the LAA wall while avoiding the left atrial body, intracavitary flow signals, and adjacent tissue. The software generated segmental strain curves and a color-coded strain map. The vertical yellow lines mark consecutive R waves on the simultaneous ECG, which define the analyzed cardiac cycle and the zero-strain reference. **(B)** Measurement of LAA-SLE. In a TEE view displaying the LAA long axis, the M-mode cursor was aligned along this axis; the double-headed arrow indicates the maximal longitudinal excursion of the LAA wall during one cardiac cycle. **(C)** Measurement of LAA-s′. Using tissue Doppler imaging with the sample volume aligned parallel to the LAA long axis, the arrow indicates the peak tissue velocity during the LAA contraction-related phase. 2D-STE, two-dimensional speckle-tracking echocardiography; LAA, left atrial appendage; LAA-GLS, left atrial appendage global longitudinal strain; LAA-SLE, left atrial appendage systolic longitudinal excursion; LAA-s′, left atrial appendage peak systolic tissue velocity; ROI, region of interest; TEE, transesophageal echocardiography.

#### 2.4.4 LAA structure–function coupling index (LAA-SFCI)

In this study, LAA-SFCI was defined as a family of composite indices reflecting the coupling between LAA structural remodeling burden and functional status, rather than a single fixed formula. Specifically, LAA-VI served as the structural component and was paired with different LAA functional parameters to construct multiple candidate LAA-SFCIs: LAA-VI/LAA-PEV, LAA-VI/LAA-PFV, LAA-VI/LAA-EF, LAA-VI/LAA-SLE, LAA-VI/LAA-s′, LAA-VI/LAA-FAC, and LAA-VI/LAA-GLS.

### 2.5 Ethics

The study protocol was approved by the institutional ethics committee of our center (Approval No. IEC-K-AF-016-1.2) and was conducted in accordance with the principles of the Declaration of Helsinki. Written informed consent was obtained from all participants before examination and collection of study data. All clinical and imaging data were de-identified prior to analysis to protect patient privacy.

## 3 Statistical analysis

Continuous variables are presented as mean ± SD and categorical variables as *n* (%). Across the four groups, continuous variables were compared using one-way ANOVA or the Kruskal–Wallis test and categorical variables using the *χ*^2^ or Fisher exact test; the trend in the structural component LAA-VI across the ordered AF stages was assessed using the Jonckheere–Terpstra test. With AF stage as an ordinal dependent variable, ordinal logistic regression was used to assess the association of each candidate LAA-SFCI with higher stage; each index was standardized and entered separately, and *β*, the odds ratio per 1-SD (OR per 1-SD), 95% CI, and the Akaike information criterion (AIC) were reported, with the principal LAA-SFCI determined by AIC. For the principal LAA-SFCI (LAA-VI/LAA-GLS), stepwise-adjusted models were constructed to evaluate its independent association, with collinearity assessed by the variance inflation factor and the proportional-odds assumption assessed with the Brant test (not estimable for the model including rhythm during TEE, because all Stage 1–2 patients were in sinus rhythm); when the assumption was not met, cutpoint-specific cumulative logistic regressions (Stage ≥2 vs. Stage 1, Stage ≥3a vs. Stage ≤2, and Stage 3b vs. Stage ≤3a) were fitted as a sensitivity analysis relaxing this assumption; because the assumption was not met for the untransformed index but was met after natural-log transformation, the standardized log-transformed index (per 1-SD) was used as the primary predictor in all ordinal models, and the untransformed index (per 0.1-unit) is reported for clinical interpretation; because a ratio with strain in the denominator may be disproportionately affected by very low strain values, the distributions of LAA-GLS and LAA-VI/LAA-GLS were examined for near-zero strain values and extreme values (> Q3 + 3 × IQR); extreme values were verified against the recorded LAA-VI and LAA-GLS measurements and retained, and sensitivity analyses were performed after excluding them and after winsorization at the 1st and 99th percentiles; among patients with clinical AF (Stage 3a and 3b), LAA structural parameters, functional parameters, and LAA-SFCI were compared by rhythm status during TEE. ROC curve analysis was used to evaluate the discriminative ability of the principal LAA-SFCI across AF stage comparisons, with optimal cutoffs determined by the Youden index. AUCs of LAA-VI/LAA-GLS were compared with those of LAA-VI, LAA-GLS, and LAVI in the same patients using the DeLong test for correlated ROC curves. For Stage 1–2 versus Stage 3a–3b, incremental discrimination was further assessed with the category-free net reclassification improvement (NRI) and the integrated discrimination improvement (IDI) derived from logistic regression models, with 95% CIs estimated from 1,000 bootstrap resamples. Internal validation was performed with 1,000 bootstrap resamples; in each resample, the principal index was reselected from the seven candidates by AIC and its Youden-derived cutoff was re-estimated, and optimism was calculated as the mean difference between performance in the bootstrap sample and in the original cohort. In 30 randomly selected cases, intraobserver agreement was assessed from repeated measurements by the same investigator after an interval and interobserver agreement from independent measurements by two investigators, both using ICC (2,1) (Supplementary Table S3). To assess potential spectrum bias related to the indication for TEE, LAA-VI/LAA-GLS was compared within Stages 1–2 between patients with and without prior stroke, TIA, or thromboembolism and between those with and without PFO using the Mann–Whitney U test. To explore risk re-stratification among patients without documented AF (Stages 1–2), these patients were dichotomized by the exploratory cutoff of LAA-VI/LAA-GLS for Stage 1–2 versus Stage 3a–3b (0.460 (mL/m^2^)/%), and clinical and echocardiographic characteristics were compared between those above and below this value using the Mann–Whitney U test or Fisher exact test. Two-sided *P* < 0.05 was considered statistically significant; analyses were performed using R 4.3.2 (R Foundation for Statistical Computing, Vienna, Austria).

## 4 Results

### 4.1 Baseline clinical characteristics and changes in LAA structure–function parameters across AF disease stages

A total of 267 patients were included and classified into four AF disease stages (Stage 1, 2, 3a, and 3b; Figure 1, Table 1). Baseline demographics and comorbidities were comparable across the four groups, whereas age, prior stroke, and the CHA₂DS₂-VASc score increased with advancing stage (all *P* ≤ 0.05); migraine, the most common reason for suspected PFO in Stages 1–2, was present in 82.35% and 81.54% of patients in Stages 1 and 2 and in none in Stages 3a and 3b (*P* < 0.001); the CHA₂DS₂-VASc score rose from 1.74 ± 1.03 to 2.47 ± 1.11 (*P* = 0.003). Markers of LAA blood stasis tracked disease stage: spontaneous echo contrast severity increased progressively, from grade 0 in all Stage 1 patients to grade ≥2 in all Stage 3b patients (*P* < 0.001), whereas LAA thrombus was observed only in the clinical-AF stages (3.03% and 7.35% in Stage 3a and 3b, respectively; Table 1). Prior stroke, TIA, or thromboembolism was documented in 17.65%, 18.46%, 18.18%, and 36.76% of patients in Stages 1, 2, 3a, and 3b, respectively (*P* = 0.018), and PFO in 25.00%, 16.92%, 22.73%, and 14.71% (*P* = 0.398) (Table 1). Within Stages 1–2, LAA-VI/LAA-GLS did not differ significantly between patients with and without prior stroke, TIA, or thromboembolism (0.40 ± 0.17 vs. 0.37 ± 0.12 (mL/m^2^)/%, *P* = 0.456) or between those with and without PFO (0.33 ± 0.09 vs. 0.39 ± 0.14 (mL/m^2^)/%, *P* = 0.144). On conventional TTE, LAD and LAVI were higher and LVEF was lower in the more advanced stages. Among TEE-derived parameters, the LAA structural indices (maximum LAA ostial area, LAA-EDV, LAA-ESV and LAA-VI) increased across stages; LAA-VI, for example, rose from 4.73 ± 1.34 mL/m^2^ in Stage 1 to 6.28 ± 1.63 mL/m^2^ in Stage 3b (*P* < 0.001). The LAA functional indices, including LAA-PEV, LAA-PFV, LAA-EF, LAA-SLE, LAA-s′, LAA-FAC, and LAA-GLS, also differed significantly among the groups (all *P* < 0.05) (Table 2). Overall, more advanced AF stage was associated with larger LAA volume and lower LAA mechanical function (Figure 3).

**Figure 3:**
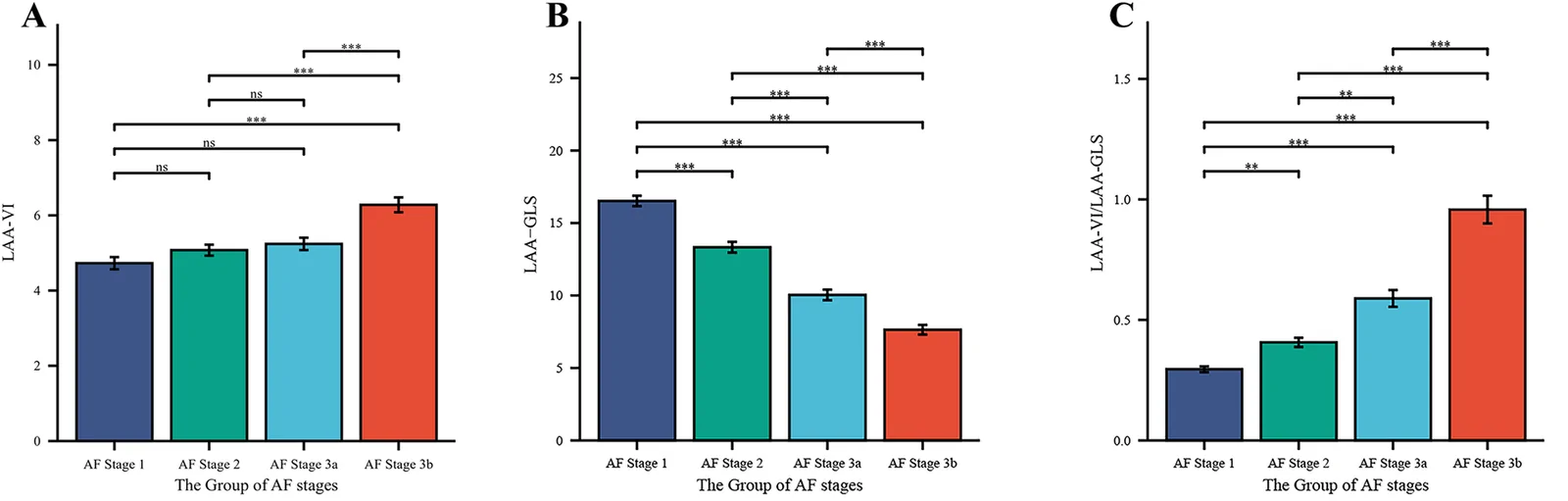
Changes in core LAA structure–function parameters across AF disease stages. **(A)** LAA-VI was higher at more advanced AF stages, reflecting a greater volumetric burden. **(B)** LAA-GLS was lower at more advanced AF stages, reflecting impaired longitudinal deformation. **(C)** LAA-VI/LAA-GLS increased across higher AF stages, reflecting greater structure–function uncoupling. Data are mean ± SEM; brackets indicate pairwise comparisons. ns, not significant; *P < 0.05; **P < 0.01; ***P < 0.001. AF, atrial fibrillation; GLS, global longitudinal strain; LAA, left atrial appendage; LAA-VI, left atrial appendage volume index.

### 4.2 Distribution characteristics of candidate LAA-SFCIs

As the structural component of the candidate LAA-SFCIs, LAA-VI was the only BSA-indexed volumetric parameter among the measured LAA structural parameters and thus best reflected the overall LAA volumetric remodeling burden; it increased significantly across AF stages, from 4.73 ± 1.34 mL/m^2^ in Stage 1 to 6.28 ± 1.63 mL/m^2^ in Stage 3b (Jonckheere–Terpstra test for trend *P* < 0.001; Figure 3). Building on this structural component, all seven candidate LAA-SFCIs constructed by pairing LAA-VI with each LAA functional parameter differed significantly across the four AF stages (all *P* < 0.001; Table 3), and LAA-VI/LAA-GLS showed the most consistent graded increase across stages (Figure 3). In ordinal logistic regression with AF stage as the ordinal outcome, every candidate index was significantly associated with a higher stage. LAA-VI/LAA-GLS provided the best model fit, with the lowest AIC among all candidate indices (*β* per 1-SD = 2.55; OR per 1-SD = 12.84, 95% CI 7.89–20.90; AIC=558.95; *P* < 0.001; Table 4). When all candidate indices were log-transformed, LAA-VI/LAA-GLS again had the lowest AIC (536.4 vs. 568.6 for the next best index, LAA-VI/LAA-SLE). Although LAA-VI/LAA-SLE showed a numerically larger odds ratio (OR per 1-SD = 16.02, 95% CI 8.82–29.08; AIC=595.68; *P* < 0.001), its model fit was inferior to that of LAA-VI/LAA-GLS. LAA-VI/LAA-GLS was therefore selected as the principal LAA-SFCI for the subsequent analyses.

**Table 3:** Candidate LAA structure–function coupling indices across AF disease stages.

Variables	AF Stage 1 (*n* = 68)	AF Stage 2 (*n* = 65)	AF Stage 3a (*n* = 66)	AF Stage 3b (*n* = 68)	*P-* value
LAA-VI/LAA-PEV, (mL/m^2^)/(cm/s)	0.10 ± 0.05	0.11 ± 0.07	0.12 ± 0.07	0.20 ± 0.13	<0.001
LAA-VI/LAA-PFV, (mL/m^2^)/(cm/s)	0.10 ± 0.05	0.10 ± 0.05	0.11 ± 0.05	0.16 ± 0.10	<0.001
LAA-VI/LAA-EF (mL/m^2^)/%	0.16 ± 0.14	0.27 ± 0.34	0.23 ± 0.13	0.60 ± 1.08	<0.001
LAA-VI/LAA-SLE, (mL/m^2^)/mm	0.26 ± 0.10	0.34 ± 0.11	0.49 ± 0.29	0.85 ± 0.56	<0.001
LAA-VI/LAA-s′ (mL/m^2^)/(cm/s)	0.18 ± 0.08	0.26 ± 0.13	0.30 ± 0.17	0.35 ± 0.17	<0.001
LAA-VI/LAA-FAC, (mL/m^2^)/%	0.17 ± 0.05	0.20 ± 0.05	0.18 ± 0.06	0.31 ± 0.10	<0.001
LAA-VI/LAA-GLS, (mL/m^2^)/%	0.33 ± 0.11	0.42 ± 0.14	0.59 ± 0.20	0.86 ± 0.36	<0.001

Values are presented as mean ± SD. AF, atrial fibrillation; FAC, fractional area change; GLS, global longitudinal strain; LAA, left atrial appendage; LAA-FAC, left atrial appendage fractional area change; LAA-GLS, left atrial appendage global longitudinal strain; LAA-SLE, left atrial appendage systolic longitudinal excursion; LAA-PEV, left atrial appendage peak emptying velocity; LAA-PFV, left atrial appendage peak filling velocity; LAA-EF, left atrial appendage ejection fraction; LAA-s′, left atrial appendage peak systolic tissue velocity; LAA-VI, left atrial appendage volume index; SD, standard deviation; VI, volume index. LAA-GLS represents the absolute magnitude of software-derived signed LAA-GLS.

**Table 4:** Ordinal logistic regression analysis of candidate LAA structure–function coupling indices for AF disease stage.

Candidate index	*β* per 1-SD	SE	OR per 1-SD (95% CI)	z/T	AIC	BIC	*P* -value
LAA-VI/LAA-PEV	1.17	0.18	3.21 (2.25–4.59)	6.40	690.04	704.39	<0.001
LAA-VI/LAA-PFV	0.82	0.15	2.26 (1.68–3.06)	5.33	711.36	725.71	<0.001
LAA-VI/LAA-EF	1.73	0.41	5.65 (2.51–12.73)	4.18	713.03	727.38	<0.001
LAA-VI/LAA-SLE	2.77	0.30	16.02 (8.82–29.08)	9.11	595.68	610.03	<0.001
LAA-VI/LAA-s′	0.77	0.12	2.17 (1.70–2.77)	6.20	703.90	718.25	<0.001
LAA-VI/LAA-FAC	1.34	0.16	3.89 (2.85–5.31)	8.55	651.04	665.39	<0.001
LAA-VI/LAA-GLS	2.55	0.25	12.84 (7.89–20.90)	10.27	558.95	573.30	<0.001

AF, atrial fibrillation; AIC, Akaike information criterion; BIC, Bayesian information criterion; CI, confidence interval; FAC, fractional area change; GLS, global longitudinal strain; LAA, left atrial appendage; LAA-FAC, left atrial appendage fractional area change; LAA-GLS, left atrial appendage global longitudinal strain; LAA-SLE, left atrial appendage systolic longitudinal excursion; LAA-PEV, left atrial appendage peak emptying velocity; LAA-PFV, left atrial appendage peak filling velocity; LAA-EF, left atrial appendage emptying fraction; LAA-s′, left atrial appendage peak systolic tissue velocity; LAA-VI, left atrial appendage volume index; OR, odds ratio; SD, standard deviation; SE, standard error; VI, volume index. LAA-GLS represents the absolute magnitude of software-derived signed LAA-GLS.

### 4.3 Discriminative performance of the principal LAA-SFCI (LAA-VI/LAA-GLS) across AF stage comparisons

ROC curve analysis showed that the principal LAA-SFCI (LAA-VI/LAA-GLS) discriminated between adjacent points along the AF disease continuum and had the highest AUC in each comparison compared with the structural parameter (LAA-VI) and the functional parameter (LAA-GLS) (Figure 4). For Stage 1 versus Stage 2, representing early atrial remodeling, the AUC of LAA-VI/LAA-GLS was 0.751, exceeding that of LAA-VI (0.583) and LAA-GLS (0.670). For Stage 3a versus Stage 3b, the AUC was 0.764, exceeding that of LAA-VI (0.679) and LAA-GLS (0.613). The highest discriminative performance was observed for Stage 1–2 versus Stage 3a–3b, corresponding to the distinction between patients without documented AF and those with clinical AF, with an AUC of 0.904, exceeding that of LAA-VI and LAA-GLS; the exploratory optimal cutoff was 0.460 (mL/m^2^)/%, with sensitivity, specificity, and accuracy of 82.1%, 86.5%, and 84.3% (Supplementary Table S4). In paired DeLong comparisons, the AUC of LAA-VI/LAA-GLS for Stage 1–2 versus Stage 3a–3b was significantly higher than that of LAA-GLS (*Δ*AUC 0.081, 95% CI 0.040–0.121), LAA-VI (*Δ*AUC 0.244, 95% CI 0.181–0.307), and LAVI (AUC 0.621; *Δ*AUC 0.283, 95% CI 0.206–0.360) (all *P* < 0.001). For Stage 1 versus Stage 2, the AUC of the index was significantly higher than that of LAA-VI (*P* = 0.001), whereas the differences from LAA-GLS (*P* = 0.068) and LAVI (*P* = 0.223) were not significant; for Stage 3a versus Stage 3b, it was significantly higher than those of LAA-GLS (*P* < 0.001) and LAVI (*P* = 0.023) but not LAA-VI (*P* = 0.136) (Supplementary Table S6). For Stage 1–2 versus Stage 3a–3b, replacing LAA-GLS with LAA-VI/LAA-GLS yielded a category-free NRI of 0.56 (95% CI 0.27–0.97) and an IDI of 0.15 (95% CI 0.07–0.24), and adding the index to LAVI yielded an NRI of 1.22 (95% CI 1.07–1.47) and an IDI of 0.44 (95% CI 0.33–0.55) (all *P* < 0.01; Supplementary Table S7). In bootstrap internal validation, LAA-VI/LAA-GLS was reselected as the principal index in 84.2% of resamples; the optimism in AUC was 0.004, yielding an optimism-corrected AUC of 0.900, with corrected sensitivity, specificity, and accuracy of 80.4%, 85.0%, and 82.7%, respectively. The bootstrap median cutoff was 0.455 (95% interval 0.396–0.472) (mL/m^2^)/% (Supplementary Table S12).

**Figure 4:**
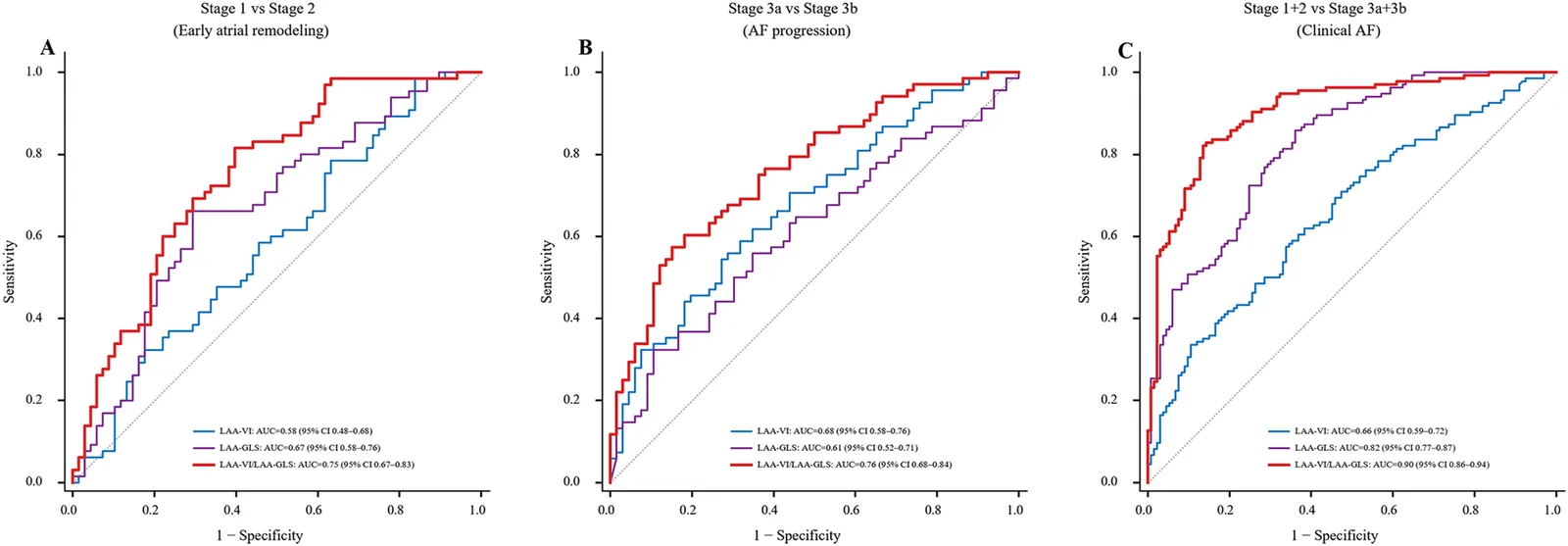
Discriminative performance of LAA-VI, LAA-GLS, and LAA-VI/LAA-GLS across key AF disease-stage comparisons. ROC curves comparing the structural parameter (LAA-VI), the functional parameter (LAA-GLS), and the principal coupling index (LAA-VI/LAA-GLS) for three AF disease-stage comparisons. **(A)** Stage 1 vs. Stage 2 (early atrial remodeling): AUC=0.58, 0.67, and 0.75, respectively. **(B)** Stage 3a vs. Stage 3b (paroxysmal vs. persistent AF): AUC=0.68, 0.61, and 0.76. **(C)** Stage 1 + 2 vs. Stage 3a + 3b (clinical AF): AUC=0.66, 0.82, and 0.90. LAA-VI/LAA-GLS had the highest AUC in all three comparisons; for Stage 1 + 2 vs. Stage 3a + 3b, its AUC was significantly higher than those of LAA-VI and LAA-GLS (both DeLong *P* < 0.001). Pairwise DeLong comparisons for all panels are provided in Supplementary Table S6. AF, atrial fibrillation; AUC, area under the receiver operating characteristic curve; LAA, left atrial appendage; LAA-GLS, left atrial appendage global longitudinal strain; LAA-VI, left atrial appendage volume index.

### 4.4 Independence and rhythm-robustness of the LAA-VI/LAA-GLS association with AF stage

Stepwise adjusted ordinal logistic regression models showed a stable association between the principal LAA-SFCI and higher AF stage (Supplementary Table S5). Because the proportional-odds assumption was not met for the untransformed index (see below), the natural-log–transformed index was used as the primary predictor. In the unadjusted model, each 1-SD increase in the log-transformed index was associated with higher AF stage (OR=9.77, 95% CI 6.61–14.46, AIC=536.4, *P* < 0.001). After adjustment for age, sex, BMI, LVEF, and LAVI, the association remained significant (OR=11.04, 95% CI 7.17–16.98, AIC=472.8, *P* < 0.001), and it also remained significant after further adjustment for rhythm status during TEE (OR=9.01, 95% CI 5.82–13.95, AIC=439.0, *P* < 0.001) and in the sinus-rhythm-only sensitivity analysis (OR=8.03, 95% CI 5.02–12.86, AIC=368.4, *P* < 0.001). The proportional-odds assumption was met for the log-transformed index in Model 1 (Brant *χ*^2^ = 2.44, df = 2, *P* = 0.296), Model 2 (*χ*^2^ = 2.78, df = 2, *P* = 0.249; omnibus *P* = 0.068), and the sinus-rhythm subgroup (*χ*^2^ = 0.03, df = 2, *P* = 0.986), cutpoint-specific ORs were similar across thresholds (11.31, 12.47, and 7.40 in Model 1; Supplementary Table S8), and model fit was better than that of the untransformed index in every model. For clinical interpretation, the corresponding ORs per 0.1-unit increase in the untransformed index were 2.33 (95% CI 1.98–2.74), 2.39 (95% CI 2.01–2.84), 2.16 (95% CI 1.82–2.56), and 2.25 (95% CI 1.83–2.78) (Supplementary Table S5). The Brant test indicated a departure from the proportional-odds assumption for the untransformed index in Model 1 (*χ*^2^ = 12.45, df = 2, *P* = 0.002) and Model 2 (*P* = 0.003), but not in the sinus-rhythm subgroup (*χ*^2^ = 2.67, df = 2, *P* = 0.263). In cutpoint-specific logistic regressions relaxing this assumption, the index remained significantly associated with higher stage at every threshold, with ORs per 0.1-unit of 3.46 (95% CI 2.36–5.06) for Stage ≥2 versus Stage 1, 2.82 (95% CI 2.18–3.64) for Stage ≥3a versus Stage ≤2, and 1.89 (95% CI 1.60–2.23) for Stage 3b versus Stage ≤3a (all *P* < 0.001), with similar estimates after adjustment (Supplementary Table S8). Thus, the direction and significance of the association were consistent across thresholds, although its magnitude was larger at earlier thresholds; the common OR for the untransformed index should therefore be interpreted only as a summary estimate. The lowest absolute LAA-GLS value was 3.0%, and no near-zero strain values were observed. LAA-VI/LAA-GLS was right-skewed (skewness 1.85; median 0.45, IQR 0.35–0.67 (mL/m^2^)/%), with two extreme values, both in Stage 3b. Excluding these values or winsorizing the index did not materially change the association (OR per 0.1-unit 2.33, 95% CI 1.98–2.74) or the AUC for Stage 1–2 versus Stage 3a–3b (0.903–0.904). After natural-log transformation (skewness 0.44), the distribution of the index was approximately symmetric; because ROC analysis is invariant to monotonic transformation, the AUC was unchanged (Supplementary Table S9).

Among patients with clinical AF (Stage 3a and 3b), subgroup analysis by rhythm status during TEE showed that patients in AF rhythm had higher LAA-VI than those not in AF rhythm, whereas LAA-VI/LAA-GLS did not differ significantly between rhythm groups (Table 5). In Stage 3a, patients in AF rhythm had higher LAA-VI (5.89 ± 1.18 vs. 4.35 ± 0.96 mL/m^2^, *P* < 0.001) and higher LAA-GLS (10.31 ± 2.80% vs. 8.76 ± 2.91%, *P* = 0.036), whereas LAA-VI/LAA-GLS did not differ significantly (0.62 ± 0.20 vs. 0.55 ± 0.19, *P* = 0.152). In Stage 3b, patients in AF rhythm again had higher LAA-VI (6.77 ± 1.52 vs. 5.33 ± 1.34 mL/m^2^, *P* < 0.001), whereas LAA-GLS (8.86 ± 3.23% vs. 7.41 ± 3.68%, *P* = 0.105) and LAA-VI/LAA-GLS (0.86 ± 0.35 vs. 0.87 ± 0.36, *P* = 0.897) did not differ significantly between rhythm groups (Table 5).

**Table 5:** Effect of AF rhythm during TEE on LAA-VI/LAA-GLS in patients with clinical AF.

Variables	AF Stage 3a (*n* = 66)		*P*-value	AF Stage 3b (*n* = 68)		*P*-value
	Non-AF rhythm (*n* = 28)	AF rhythm (*n* = 38)		Non-AF rhythm (*n* = 23)	AF rhythm (*n* = 45)	
LAA-VI, mL/m^2^	4.35 ± 0.96	5.89 ± 1.18	<0.001	5.33 ± 1.34	6.77 ± 1.52	<0.001
LAA-GLS, %	8.76 ± 2.91	10.31 ± 2.80	0.036	7.41 ± 3.68	8.86 ± 3.23	0.105
LAA-VI/LAA-GLS, (mL/m^2^)/%	0.55 ± 0.19	0.62 ± 0.20	0.152	0.87 ± 0.36	0.86 ± 0.35	0.897

Values are presented as mean ± SD. SD, standard deviation; AF, atrial fibrillation; GLS, global longitudinal strain; LAA, left atrial appendage; LAA-VI, left atrial appendage volume index; TEE, transesophageal echocardiography.

### 4.5 LAA-VI/LAA-GLS among patients without documented AF

Among the 133 patients without documented AF, 18 (13.5%) had LAA-VI/LAA-GLS above 0.460 (mL/m^2^)/%, the exploratory cutoff distinguishing Stage 1–2 from Stage 3a–3b; these included 4 of 68 (5.9%) Stage 1 and 14 of 65 (21.5%) Stage 2 patients (Supplementary Table S10). Compared with the remaining patients, this subgroup had a higher LAA-VI (5.50 ± 1.08 vs. 4.80 ± 1.28 mL/m^2^, *P* = 0.026), a lower LAA-GLS (9.21 ± 2.30% vs. 14.54 ± 3.45%, *P* < 0.001), a higher prevalence of coronary heart disease (27.8% vs. 8.7%, *P* = 0.033), and a numerically higher CHA₂DS₂-VASc score (2.44 ± 1.10 vs. 1.87 ± 1.15, *P* = 0.052). SEC was more frequent in this subgroup (66.7% vs. 37.4%, *P* = 0.023), although this difference largely reflected its higher proportion of Stage 2 patients and was not observed within Stage 2 alone. In contrast, LAVI (36.24 ± 4.86 vs. 38.06 ± 4.00 mL/m^2^, *P* = 0.210), left atrial diameter, LVEF, age, and the prevalence of prior stroke, TIA, or thromboembolism (22.2% vs. 17.4%, *P* = 0.741) did not differ significantly between the two groups. The proportion of patients above the cutoff was 21.4% among those with LAVI ≤ 34 mL/m^2^ and 11.4% among those with LAVI > 34 mL/m^2^. In a sensitivity analysis in which the 50 Stage 1 patients with LAVI > 34 mL/m^2^ were reclassified as Stage 2 in accordance with the guideline definition, the association of LAA-VI/LAA-GLS with higher AF stage was maintained (log-transformed index: OR per 1-SD 7.59, 95% CI 5.26–10.97; adjusted OR 9.18, 95% CI 6.00–14.05; untransformed index: OR per 0.1-unit 2.18, 95% CI 1.86–2.54; adjusted OR 2.30, 95% CI 1.94–2.74), and the AUC for Stage 1–2 versus Stage 3a–3b was unchanged (0.904), whereas discrimination between Stage 1 and Stage 2 was weaker (AUC 0.621, 95% CI 0.476–0.766) (Supplementary Table S11). Excluding the 7 Stage 1 patients whose only risk factor was advanced age did not change the results (log-transformed index: OR per 1-SD 10.38, 95% CI 6.93–15.55; untransformed index: OR per 0.1-unit 2.38, 95% CI 2.02–2.81; AUC for Stage 1–2 versus Stage 3a–3b 0.907).

## 5 Discussion

### 5.1 Principal findings

In this prospectively enrolled, cross-sectional observational study, we developed and evaluated a family of TEE-derived LAA-SFCIs. LAA-VI/LAA-GLS was selected as the principal index because its corresponding model yielded the lowest AIC. This ratio may increase because of a greater LAA volumetric remodeling burden, reduced longitudinal deformation capacity, or both. A higher value therefore represents a greater volumetric remodeling burden per unit of longitudinal deformation capacity, reflecting a more pronounced imbalance between LAA structural burden and functional status.

The principal findings were as follows. First, across higher AF stages, LAA-VI increased, LAA-GLS decreased, and LAA-VI/LAA-GLS rose across stages, consistent with stage-related LAA structure–function uncoupling. Second, the index remained significantly associated with higher AF stage after adjustment for demographic characteristics, conventional echocardiographic parameters, and rhythm during TEE, with consistent findings in the sinus-rhythm subgroup. Third, LAA-VI/LAA-GLS had the highest AUC across the examined stage comparisons, with the greatest discrimination between Stage 1–2 and Stage 3a–3b, where its AUC was significantly higher than those of LAA-VI, LAA-GLS, and LAVI (all DeLong *P* < 0.001). These findings suggest that integrating LAA volumetric remodeling burden with longitudinal mechanical deformation may provide complementary information for characterizing stage-related LAA remodeling across the AF continuum. Given the cross-sectional design, however, these findings reflect associations across AF stages rather than within-patient longitudinal progression or prognostic value.

### 5.2 Imaging and pathophysiological interpretation of LAA-SFCI across the AF disease continuum

The LAA remodeling observed in this study was not characterized by parallel deterioration across all structural and functional parameters but was instead dominated by an increased volumetric burden and reduced longitudinal mechanical function. Across groups, LAA-VI increased from 4.73 to 6.28 mL/m^2^, whereas LAA-GLS decreased from 14.90% to 8.37% (both *P* < 0.001 across groups), and LAA-SLE showed a similar overall direction of change. LAA end-systolic volume increased, and LAA-EF decreased across stages (both *P* < 0.001), consistent with the overall trend of increasing volumetric burden and declining emptying function. Although emptying velocity and fractional area change fluctuated across AF stages, tissue Doppler s’ generally decreased with advancing AF stage but remained nearly unchanged between Stages 2 and 3a; therefore, it did not exhibit a strictly monotonic pattern. These findings suggest that different imaging parameters may capture distinct facets of LAA remodeling: volumetric measures primarily reflect the volumetric remodeling burden, longitudinal strain reflects the mechanical deformation capacity of the LAA wall, and flow-velocity and global emptying indices may additionally be influenced by cardiac rhythm during examination, heart rate, and instantaneous loading conditions. With regard to the sampling site, LAA-SLE and LAA-s′ in this study were both measured at the LAA tip: the LAA ostium is bordered anteromedially by the aortic root and inferiorly by the mitral annulus, so measurements at the ostium are readily affected by passive tethering from aortic pulsation and mitral annular motion, whereas the proximal region adjacent to the coumadin (warfarin) ridge is relatively fixed and has limited excursion; the tip, in contrast, is the most mobile, free-moving segment, and its systolic displacement toward the ostial plane more faithfully reflects the intrinsic longitudinal shortening of the LAA, thereby distinguishing the mechanical function of the LAA from the passive motion of adjacent structures and allowing these two parameters to more reliably characterize the longitudinal mechanical state of the LAA. Taken together, these parameter characteristics indicate that LAA remodeling across AF stages is manifested primarily as an imbalance between volumetric burden and longitudinal mechanical function; compared with any single morphological or emptying parameter, the combined assessment of LAA volume and longitudinal deformation is more informative for identifying this structural–functional imbalance (24, 25).

From a pathophysiological perspective, these findings may represent the LAA manifestation of progressive atrial cardiomyopathy. Hemodynamic load, atrial cardiomyocyte hypertrophy, interstitial fibrosis, inflammation, and extracellular matrix remodeling can reduce atrial wall compliance and contractile coordination while promoting atrial enlargement and mechanical dysfunction (26, 27). Given its thin wall, complex muscle bundle architecture, and high compliance, longitudinal deformation of the LAA may be particularly sensitive to local fibrosis and mechanical dyssynchrony (28, 29). After the onset of clinical AF, rapid and irregular atrial activation may further compromise coordinated LAA shortening and effective emptying. The electrical and structural remodeling associated with persistent AF may compound this impairment (30, 31). These mechanisms may explain the clearer stage-related gradients observed in LAA-GLS and LAA-SLE, as opposed to the rhythm-dependent or nonlinear patterns of some flow and global emptying indices. However, because the present study was based on cross-sectional comparisons, these mechanisms serve primarily to interpret the observed imaging associations. Our findings cannot establish whether LAA functional decline precedes volumetric enlargement or define the temporal sequence by which individuals progress from earlier to later stages.

Against this background, LAA-VI/LAA-GLS may be interpreted as a composite imaging index reflecting the relative balance between structural burden and longitudinal mechanical function. The ratio increased from 0.33 (mL/m^2^)/% in Stage 1 to 0.86 (mL/m^2^)/% in Stage 3b (*P* < 0.001). This increase may result from LAA enlargement, impaired longitudinal deformation, or both. Among the candidate combinations, the model incorporating LAA-VI/LAA-GLS yielded the lowest AIC and BIC, indicating that the combination of a volumetric measure and longitudinal strain provided the best overall fit for differences across AF stages. LAA-VI/LAA-SLE also showed a strong association with AF stage, further supporting longitudinal mechanical dysfunction as an important component of structural–functional uncoupling. Meanwhile, SEC severity generally increased with AF stage, and LAA thrombi were observed only in Stages 3a and 3b. This distribution is consistent with previous evidence linking LAA enlargement and mechanical impairment to local blood stasis (32, 33), suggesting that volumetric enlargement and impaired longitudinal deformation across AF stages may jointly constitute an imaging substrate for local blood stasis. Taken together, LAA-VI/LAA-GLS integrates volumetric burden and longitudinal mechanical function within a single assessment framework. Its variation across AF stages reflects differences in the extent of LAA structural–functional imbalance along the AF disease continuum, providing a new imaging perspective on stage-related LAA remodeling. As a ratio with strain in the denominator, the index could in principle be inflated by very low strain values; however, no near-zero LAA-GLS values were observed in this cohort, and the findings were robust to the exclusion of extreme values and to logarithmic transformation. Because log transformation also satisfied the proportional-odds assumption and improved model fit, the log-transformed index was used for regression inference, whereas the untransformed ratio was retained for description, ROC analysis, and the cutoff because it is easier to calculate and interpret.

### 5.3 Incremental value of structure–function coupling indices compared with single structural or functional parameters

When assessing LAA remodeling across the AF disease continuum, structural and functional parameters provide distinct but noninterchangeable information. As a BSA-indexed measure, LAA-VI quantifies the burden of LAA volumetric remodeling, but similar LAA-VI values may correspond to different levels of longitudinal deformation. LAA-GLS reflects global longitudinal deformation of the LAA wall but does not convey the structural context underlying this functional state. In other words, a single parameter may indicate whether the LAA is enlarged or longitudinal deformation is impaired. However, it cannot determine whether mechanical function is commensurate with the corresponding volumetric burden. The value of LAA-SFCI lies in assessing the relative relationship between these two dimensions and identifying imbalances that may be overlooked when structure or function is considered alone.

Our findings indicate that the information provided by LAA-VI/LAA-GLS cannot be explained solely by global cardiac structural or functional status. The index correlated only weakly with LAVI and LVEF (r = 0.12 and −0.24, respectively), and its VIF in the multivariable model was 1.11. After further adjustment for age, sex, BMI, LVEF, LAVI, and rhythm during TEE, its association with higher AF stage remained significant. This association was also consistent in the sinus-rhythm subgroup.

Furthermore, the relative discriminative performance of the single structural and functional parameters was not consistent across all stage comparisons. For Stage 1 versus Stage 2 and Stage 1–2 versus Stage 3a–3b, the AUC of LAA-GLS was numerically higher than that of LAA-VI. By contrast, for Stage 3a versus Stage 3b, LAA-VI had a numerically higher AUC than LAA-GLS. This variation suggests that the relative contributions of structural and functional information may differ across stage comparisons (34). In contrast, LAA-VI/LAA-GLS had the highest AUC in all three comparisons (0.751, 0.764, and 0.904, respectively), although its advantage over the better-performing single parameter reached statistical significance only for Stage 1–2 versus Stage 3a–3b. For this comparison, its AUC was significantly higher than those of LAA-VI (0.661) and LAA-GLS (0.824) (both DeLong *P* < 0.001), and its incremental discrimination was supported by the NRI and IDI. Because these estimates were obtained in the derivation cohort, they should be regarded as exploratory. Bootstrap internal validation indicated minimal optimism (optimism-corrected AUC 0.900) and a stable cutoff; however, internal validation cannot replace external validation in independent cohorts. Thus, the advantage of the coupling index does not depend on poor performance of either single parameter. Rather, when the relative performance of structural and functional parameters varies across stage comparisons, the index provides a more consistent integrated characterization of their relationship.

### 5.4 Clinical implications of LAA-SFCI along the AF disease continuum

From a clinical perspective, the value of LAA-VI/LAA-GLS lies not in diagnosing or staging AF, which is established by clinical history and electrocardiographic monitoring, but in characterizing the local LAA substrate along the AF disease continuum (35). In this study, the comparison of Stage 1–2 with Stage 3a–3b, which corresponds to the distinction between the at-risk or pre-AF stages and clinical AF in the 2023 ACC/AHA/ACCP/HRS guideline (2), was used to define the LAA phenotype associated with clinical AF (AUC 0.904; exploratory cutoff 0.460 (mL/m^2^)/%), rather than to propose a test for known AF. Applying this reference to patients without documented AF identified a subgroup of 13.5% whose LAA phenotype resembled that of clinical AF. These patients could not be distinguished by LAVI, left atrial diameter, LVEF, or age, and 21.4% of patients with a normal LAVI exceeded the cutoff, suggesting that the index captures local LAA remodeling that is not reflected by conventional measures of atrial size. In patients undergoing TEE for suspected PFO, whether after a cerebrovascular event or because of migraine, such a phenotype might identify candidates for intensified rhythm monitoring to detect subclinical AF, consistent with previous evidence that LAA strain is associated with subclinical AF in embolic stroke of undetermined source (14) and that left atrial mechanical dysfunction is associated with ischemic stroke in people without prevalent AF (35). However, because the cutoff was derived from the same cohort and no follow-up data were available, this subgroup should be regarded as hypothesis-generating rather than as a validated high-risk category. As the index relies on TEE, its potential application is limited to patients with an established clinical indication for TEE and does not extend to AF screening in the general population.

Rhythm subgroup analyses provided further context for interpreting the index under actual TEE examination conditions. In both Stage 3a and Stage 3b, LAA-VI was significantly higher among patients examined during AF than among those examined during sinus rhythm. The corresponding values were 5.89 vs. 4.35 mL/m^2^ and 6.77 vs. 5.33 mL/m^2^, respectively (both *P* < 0.001). LAA-GLS values were also numerically higher among patients examined during AF. Because examination rhythm was associated with changes in both the numerator and denominator, these changes may have partially offset each other within the ratio. Consequently, LAA-VI/LAA-GLS did not differ significantly between the two rhythm groups in either Stage 3a (0.62 vs. 0.55 (mL/m^2^)/%, *P* = 0.152) or Stage 3b (0.86 vs. 0.87 (mL/m^2^)/%, *P* = 0.897). These findings suggest that LAA-VI/LAA-GLS may have some relative stability against rhythm-related variation. Nevertheless, the absence of significant between-group differences does not establish complete independence from examination rhythm, particularly because rhythm was associated with both component measurements (35, 36). Future studies and clinical reports should therefore provide the rhythm at TEE together with the original LAA-VI and LAA-GLS measurements to avoid interpreting the ratio in isolation.

Accordingly, among patients undergoing TEE, LAA-VI/LAA-GLS is more appropriately regarded as a composite imaging phenotype of the relative imbalance between local LAA volumetric burden and longitudinal mechanical function than as a stand-alone tool for diagnosing AF or predicting thromboembolic risk. A higher ratio indicates a greater volumetric burden per unit of longitudinal deformation. Its clinical interpretation should incorporate medical history, standard or ambulatory electrocardiographic findings, rhythm at TEE, LAVI, the CHA₂DS₂-VASc score, and TEE findings such as SEC and LAA thrombus.

Beyond this role, the pathophysiological basis of the index suggests several clinical questions for future investigation. First, LAA dilatation and impaired deformation are established substrates of blood stasis (32, 33), and in the present cohort SEC severity and LAA thrombus were concentrated in the higher stages; whether LAA-VI/LAA-GLS predicts LAA thrombus or SEC independently of LAA emptying velocity and clinical risk scores requires dedicated studies, as only 7 patients in this cohort had LAA thrombus. Second, LAA mechanical function has been associated with AF recurrence after catheter ablation (24), suggesting that the index could be evaluated as a pre-procedural marker of recurrence risk. Third, because thromboembolic risk in patients without documented AF is currently assessed mainly by clinical scores, an imaging marker of local LAA remodeling might help refine rhythm monitoring or, ultimately, anticoagulation decisions in selected patients, particularly those with cryptogenic stroke. None of these applications was tested in the present cross-sectional study; the index should not currently be used to guide anticoagulation therapy or predict clinical outcomes, and each potential application requires prospective validation with longitudinal follow-up.

Left atrial reservoir strain (LASr) provides a simpler, noninvasive TTE-based marker of atrial mechanical dysfunction that is relevant to LAA thrombogenicity. In 115 patients with nonvalvular AF scheduled for electrical cardioversion, LASr correlated strongly with TEE-derived LAA global strain (r = 0.81) and was the only independent correlate of LAA thrombosis, defined as thrombus or grade 4 SEC (OR 0.40, 95% CI 0.24–0.70), with an LASr ≤ 9.3% showing excellent discrimination (AUC 0.98) (37). These findings support the concept that global left atrial reservoir dysfunction may mirror LAA mechanical impairment. Rather than competing with LASr, LAA-SFCI may represent a complementary level of atrial phenotyping: LASr as an accessible marker of global atrial cardiomyopathy that can be obtained in any patient, and LAA-SFCI as a more detailed characterization of local appendage structure–function uncoupling in patients already undergoing clinically indicated TEE. Because the discrimination reported for LASr refers to LAA thrombosis in patients with established AF, it is not directly comparable with the AF-stage discrimination of LAA-SFCI in the present study. LASr was not measured in this study, so a head-to-head comparison was not possible; whether LAA-SFCI provides information incremental to LASr should be addressed in future studies.

## 6 Limitations

This study has several limitations. First, it was a single-center, cross-sectional study. Second, all participants underwent TEE for clinical indications. Patients in Stage 1 and Stage 2 were not healthy controls; all were referred for TEE to evaluate suspected patent foramen ovale, mostly because of migraine and less often after a prior stroke, TIA, or thromboembolism. Therefore, Stage 1 and Stage 2 patients represent a referral population with suspected PFO rather than an unselected at-risk or pre-AF population, and spectrum bias related to the indication for TEE cannot be excluded. Comorbidities underlying these indications may themselves be associated with atrial remodeling and could have increased LAA volume or reduced LAA strain in the earlier stages; such bias may have influenced the differences between Stages 1–2 and Stages 3a–3b, although its direction and magnitude cannot be determined. Migraine, present in most patients in Stages 1–2 but absent in Stages 3a–3b, may itself be associated with atrial remodeling, and its effect on the comparison between these groups likewise cannot be determined. Within Stages 1–2, LAA-VI/LAA-GLS did not differ significantly according to prior stroke, TIA, or thromboembolism or the presence of PFO, although a nonsignificant trend toward higher values was observed among Stage 2 patients with prior thromboembolic events (0.50 ± 0.21 vs. 0.41 ± 0.11 (mL/m^2^)/%, *P* = 0.057). Nevertheless, the absolute values and cutoffs reported here may not be generalizable to unselected populations. In addition, Stage 2 was defined by Holter-detected atrial electrical abnormalities, and left atrial enlargement was not used as a staging criterion; therefore, Stage 1 included patients with an enlarged left atrium, and the distinction between Stage 1 and Stage 2 differs from the full guideline definition. Although the principal findings were robust to guideline-concordant reclassification, the discrimination between Stage 1 and Stage 2 should be interpreted with caution. Moreover, although the index differed significantly between stages at the group level, its values overlapped considerably between stages at the individual level (Tables 2, 3); a single measurement therefore cannot be used to assign AF stage or to guide decisions in an individual patient. Stage 1 was also heterogeneous and included 7 patients whose only risk factor was advanced age. Third, this study focused primarily on Stage 1, Stage 2, Stage 3a, and Stage 3b and did not include patients in Stage 3c, Stage 3d, or Stage 4. The applicability of LAA-SFCI in patients with more advanced AF, successful post-ablation status, or permanent AF therefore requires further evaluation. Fourth, the complex anatomy of the LAA may make volume and strain measurements susceptible to TEE imaging-plane selection, image quality, RR-interval variability, and STE software algorithms. In AF, LAA-GLS was averaged over five consecutive cycles without index-beat selection, so beat-to-beat variation related to irregular R–R intervals may have contributed to measurement variability. In addition, the exclusion of patients with inadequate image quality (*n* = 22) may have favored patients with more favorable acoustic windows, and the feasibility of LAA-STE in unselected patients may therefore be lower. In particular, LAA morphology (chicken wing, cactus, windsock, or cauliflower) was not classified, so its potential influence on volume and strain measurements could not be assessed; in complex or multilobed morphologies, parts of the LAA wall may be poorly aligned with the ultrasound beam or move out of the imaging plane during the cardiac cycle, which multiplane acquisition and averaging across four views can reduce but not eliminate. Furthermore, medication use other than anticoagulation, including antiarrhythmic, rate-control, and renin–angiotensin system–modulating drugs, was not systematically recorded, and its potential influence on atrial remodeling and LAA mechanics could not be evaluated. Furthermore, this study did not directly evaluate the independent associations of LAA-SFCI with SEC, LAA thrombus, stroke events, or other longitudinal clinical outcomes. Further multicenter studies incorporating consecutive enrollment, prospective external validation, and longitudinal follow-up are needed to confirm its measurement reproducibility, reference thresholds, stage-discrimination performance, and potential clinical value.

## 7 Conclusions

In this single-center, cross-sectional study, we developed a family of TEE-derived LAA structure–function coupling indices and identified LAA-VI/LAA-GLS as the principal index. Along the AF disease continuum, the index was higher at more advanced stages, was associated with AF stage independently of conventional clinical and echocardiographic variables, and discriminated patients with clinical AF from those without documented AF better than its individual components or LAVI. Using clinical AF as a reference, the index identified a subgroup of patients without documented AF whose LAA phenotype resembled that of clinical AF and who could not be recognized by conventional atrial size measures. Because stage differences were assessed cross-sectionally and the cutoff was derived in the same cohort, these findings do not demonstrate individual progression and should be regarded as hypothesis-generating. Prospective, multicenter studies with longitudinal follow-up are needed to determine whether LAA-SFCI predicts incident AF, LAA thrombosis, stroke, or other clinical outcomes beyond established clinical and echocardiographic predictors.

## Data Availability

The raw data supporting the conclusions of this article will be made available by the authors, without undue reservation.
