# Supplementary material for: Development and evaluation of a transesophageal echocardiography–derived left atrial appendage structure–function coupling index across the atrial fibrillation disease continuum
Source: Front Cardiovasc Med. 2026 Oct 28;13:1951089. doi: 10.3389/fcvm.2026.1951089 (PMC13751335; doi:10.3389/fcvm.2026.1951089)
Supplement: Supplementary file 1 [file Datasheet1.docx]

**Supplementary Material**

**Supplementary Methods. Sample Size Estimation**

The sample size was determined a priori for the primary endpoint—the between-group difference in left atrial appendage (LAA) structure–function parameters across the four atrial fibrillation (AF) disease stages (Stage 1, 2, 3a, and 3b)—analyzed by one-way analysis of variance (ANOVA). Because the optimal structure–function coupling index was not defined at the design stage, the sample size could not be based on the index itself. A conservative moderate effect size (Cohen’s *f* = 0.25), an established benchmark independent of the observed data, was therefore assumed a priori to avoid overestimating the effect and thereby underestimating the required sample size. The full input parameters are summarized in Table S1-S2.

**Supplementary Table S1. Input parameters for the a priori sample size calculation.**

| **Parameter** | **Value** |
| --- | --- |
| Statistical test | One-way ANOVA |
| Number of groups (k) | 4 |
| *Effect size (Cohen’s f)* | 0.25 (moderate) |
| Significance level (α, two-sided) | 0.05 |
| Statistical power (1 − β) | 0.90 |
| Allocation ratio | 1: 1: 1: 1 |
| Software | G*Power 3.1 |
| Required n per group | 58 |
| Required total N | 232 |

To assess robustness, we tabulated the required per-group sample size across a range of effect sizes (Table S2). The enrolled sample (65–68 per group) met the requirement for the assumed medium effect (Cohen's f = 0.25; 58 per group at 90% power); detecting a smaller effect (f ≤ 0.20) would have required ≥70 per group, so the design was powered for a medium or larger between-group difference.

**Supplementary Table S2. Sensitivity analysis: required per-group sample size across effect sizes (one-way ANOVA; four groups; two-sided α = 0.05).**

| ***Cohen’s f*** | **Power = 0.80** | **Power = 0.90** |
| --- | --- | --- |
| 0.15 | 123 | 159 |
| 0.20 | 70 | 90 |
| 0.25 (assumed) | 45 | 58 |
| 0.30 | 32 | 41 |
| 0.40 | 19 | 24 |

To satisfy this requirement while achieving balanced group sizes across the AF continuum, a stratified quota consecutive enrollment strategy was applied, with a preset cap of 80 patients per group. All eligible patients were consecutively screened and classified into one of the four stages; once a stratum reached the cap, enrollment into that stratum stopped, while consecutive enrollment continued for the unfilled strata until all four groups reached 80.

**Supplementary Table S3. Intraobserver and interobserver** **reproducibility of echocardiographic left atrial appendage measurements**

| **Parameter** | **Observer 1 (first)** | **Observer 1 (second)** | **Observer 2** | **Intraobserver ICC (95% CI)** | **Interobserver ICC (95% CI)** |
| --- | --- | --- | --- | --- | --- |
| LAA-EDV, mL | 8.41 ± 2.30 | 8.63 ± 2.59 | 7.12 ± 2.29 | 0.877  (0.760–0.921) | 0.821  (0.712–0.892) |
| LAA-PEV, cm/s | 43.03 ± 16.18 | 40.28 ± 17.45 | 42.76 ± 17.41 | 0.880  (0.786–0.926) | 0.792  (0.681–0.872) |
| LAA-PFV, cm/s | 48.68 ± 16.17 | 50.00 ± 18.92 | 61.37 ± 17.95 | 0.877  (0.778–0.941) | 0.811  (0.701–0.883) |
| LAA-SLE, mm | 12.06 ± 5.38 | 12.02 ± 8.34 | 13.58 ± 6.74 | 0.758  (0.619–0.844) | 0.712  (0.582–0.812) |
| LAA-s′, cm/s | 35.36 ± 19.14 | 37.07 ± 23.12 | 33.01 ± 10.74 | 0.648  (0.399–0.809) | 0.623  (0.412–0.772) |
| LAA-FAC, % | 25.36 ± 6.12 | 24.62 ± 9.94 | 29.87 ± 7.92 | 0.647  (0.435–0.773) | 0.631  (0.421–0.782) |
| LAA-GLS, % | 10.65 ± 4.00 | 10.96 ± 4.80 | 10.80 ± 4.01 | 0.758  (0.593–0.845) | 0.734  (0.582–0.834) |

Repeated measurements were performed in the same 30 subjects. For intraobserver reproducibility, the same sonographer repeated the measurements on two separate occasions; for interobserver reproducibility, a second, independent sonographer measured the same subjects, and the interobserver ICC was computed between the mean of the first sonographer's two measurements and the measurement of the second sonographer. Values are presented as mean ± SD.

Statistical analysis. Intraclass correlation coefficients (ICCs) were estimated using a two-way random-effects, absolute-agreement, single-measurement model [ICC (2,1)]. Ninety-five percent confidence intervals were estimated by case bootstrap. Both intraobserver and interobserver ICCs were significant (all *P* < 0.001; *P* values test the null hypothesis that ICC = 0).

Abbreviations: CI, confidence interval; EDV, end-diastolic volume; FAC, fractional area change; GLS, global longitudinal strain; ICC, intraclass correlation coefficient; LAA, left atrial appendage; LAA-SLE, left atrial appendage systolic longitudinal excursion; LAA-s′, left atrial appendage peak systolic tissue velocity; PEV, peak emptying velocity; PFV, peak filling velocity; SD, standard deviation.

**Supplementary Table S4. Discriminative Performance of LAA-VI/LAA-GLS for Key AF Stage Comparisons**

| **ROC endpoint** | **Comparison** | **AUC** | **Sensitivity** | **Specificity** | **Youden index** | **Optimal cutoff** | **Accuracy** |
| --- | --- | --- | --- | --- | --- | --- | --- |
| Early atrial remodeling | Stage 1 vs Stage 2 | 0.751 (0.668–0.833) | 0.815 | 0.603 | 0.418 | 0.330 | 0.707 |
| Clinical AF | Stage 1+2  vs  Stage 3a+3b | 0.904 (0.868–0.941) | 0.821 | 0.865 | 0.686 | 0.460 | 0.843 |
| AF progression | Stage 3a vs Stage 3b | 0.764 (0.685–0.844) | 0.574 | 0.848 | 0.422 | 0.740 | 0.709 |

Abbreviations: AF, atrial fibrillation; AUC, area under the receiver operating characteristic curve; ROC, receiver operating characteristic.

**Supplementary Table S5. Stepwise Adjusted Ordinal Logistic Regression Models for the Association Between LAA-VI/LAA-GLS and AF Stage**

| **Model** | **Adjustment variables** | **β** | **OR (95% CI)** | **LR χ² (index)** | ***P*** | **AIC** |
| --- | --- | --- | --- | --- | --- | --- |
| **Natural-log–transformed index, per 1-SD (primary analysis)** | | | | | | |
| Model 1 | LAA-VI/LAA-GLS | 2.28 | 9.77 (6.61–14.46) | 211.7 | <0.001 | 536.4 |
| Model 2 | LAA-VI/LAA-GLS, age, sex, BMI, LVEF, LAVI | 2.40 | 11.04 (7.17–16.98) | 193.3 | <0.001 | 472.8 |
| Model 3 | LAA-VI/LAA-GLS, age, sex, BMI, LVEF, LAVI, rhythm during TEE | 2.20 | 9.01 (5.82–13.95) | 140.9 | <0.001 | 439.0 |
| Model 4 | Sinus-rhythm subgroup (n = 184); LAA-VI/LAA-GLS only | 2.08 | 8.03 (5.02–12.86) | 111.3 | <0.001 | 368.4 |
| **Untransformed index, per 0.1-unit** | | | | | | |
| Model 1 | LAA-VI/LAA-GLS | 0.85 | 2.33 (1.98–2.74) | 189.2 | <0.001 | 558.9 |
| Model 2 | LAA-VI/LAA-GLS, age, sex, BMI, LVEF, LAVI | 0.87 | 2.39 (2.01–2.84) | 175.3 | <0.001 | 490.8 |
| Model 3 | LAA-VI/LAA-GLS, age, sex, BMI, LVEF, LAVI, rhythm during TEE | 0.77 | 2.16 (1.82–2.56) | 127.2 | <0.001 | 452.7 |
| Model 4 | Sinus-rhythm subgroup (n = 184); LAA-VI/LAA-GLS only | 0.81 | 2.25 (1.83–2.78) | 101.0 | <0.001 | 378.7 |

AF stage was modeled as an ordinal outcome (Stage 1 < 2 < 3a < 3b) using a proportional-odds model; the predictor is LAA-VI/LAA-GLS, entered as the natural-log–transformed index per 1-SD (SD of the log-transformed index = 0.475) or as the untransformed index per 0.1-unit increment (SD = 0.302), with covariates added stepwise. The log-transformed index is the primary predictor because the proportional-odds assumption was met for this form (Brant test: Model 1, *P* = 0.296; Model 2, *P* = 0.249; Model 4, *P* = 0.986); the untransformed index is shown for clinical interpretation. The proportional-odds assumption was assessed with the Brant test (untransformed index: Model 1, *P* = 0.002; Model 2, *P* = 0.003 for the index; Model 4, *P* = 0.263; not estimable for Model 3 because rhythm during TEE was constant within Stages 1–2); cutpoint-specific estimates are provided in Supplementary Table S8. LR χ² (index, df = 1) tests the index at each adjustment level; it remained significant in every model, and the OR was stable across adjustment and in the sinus-rhythm subset (range 8.03–11.04 per 1-SD of the log-transformed index and 2.16–2.39 per 0.1-unit of the untransformed index), supporting robustness to confounding and rhythm at examination. The index showed low collinearity with conventional measures (VIF = 1.11; r = 0.12 with LAVI, −0.24 with LVEF).

Model 1: (unadjusted): the crude, univariable association between LAA-VI/LAA-GLS and AF stage, establishing the baseline effect size.

Model 2: adjusted for demographic factors (age, sex, BMI) and conventional echocardiographic measures of chamber size and function (LVEF, LAVI), testing whether the index adds information beyond established structural and functional markers.

Model 3: additionally adjusted for rhythm during TEE. Because Stage 1–2 is in sinus rhythm, this adjustment is applied across all stages and should be interpreted with that structural constraint in mind.

Model 4: (sinus-rhythm subgroup, n = 184; unadjusted): the preferred rhythm-robustness check, confirming the association is not driven by rhythm at examination while avoiding adjustment for a variable partly determined by AF stage.

Abbreviations: AIC, Akaike information criterion; BMI, body mass index; CI, confidence interval; SD, standard deviation; VIF, variance inflation factor; AF, atrial fibrillation; LAA-VI, left atrial appendage volume index; LAA-GLS, left atrial appendage global longitudinal strain; LAVI, left atrial volume index; LR, likelihood ratio; LVEF, left ventricular ejection fraction; OR, odds ratio; TEE, transesophageal echocardiography.

**Supplementary Table S6. Paired DeLong comparison of the AUC of LAA-VI/LAA-GLS with those of LAA-VI, LAA-GLS, and LAVI**

| **Comparison** | **Comparator** | **AUC of LAA-VI/LAA-GLS (95% CI)** | **AUC of comparator (95% CI)** | **ΔAUC (95% CI)** | **DeLong *P*** |
| --- | --- | --- | --- | --- | --- |
| Stage 1 vs Stage 2 | LAA-VI | 0.751 (0.668–0.833) | 0.583 (0.486–0.680) | 0.168 (0.066–0.269) | 0.001 |
|  | LAA-GLS |  | 0.670 (0.578–0.763) | 0.081 (−0.006 to 0.167) | 0.068 |
|  | LAVI |  | 0.667 (0.573–0.760) | 0.084 (−0.051 to 0.219) | 0.223 |
| Stage 3a vs Stage 3b | LAA-VI | 0.764 (0.685–0.844) | 0.679 (0.589–0.769) | 0.085 (−0.027 to 0.197) | 0.136 |
|  | LAA-GLS |  | 0.613 (0.517–0.709) | 0.151 (0.083–0.220) | <0.001 |
|  | LAVI |  | 0.615 (0.518–0.712) | 0.150 (0.021–0.279) | 0.023 |
| Stage 1–2 vs Stage 3a–3b | LAA-VI | 0.904 (0.868–0.941) | 0.661 (0.596–0.725) | 0.244 (0.181–0.307) | <0.001 |
|  | LAA-GLS |  | 0.824 (0.775–0.872) | 0.081 (0.040–0.121) | <0.001 |
|  | LAVI |  | 0.621 (0.555–0.688) | 0.283 (0.206–0.360) | <0.001 |

ΔAUC = AUC of LAA-VI/LAA-GLS minus AUC of the comparator, estimated in the same patients. 95% CIs of AUCs and ΔAUC were derived from the DeLong variance. Abbreviations: AUC, area under the receiver operating characteristic curve; CI, confidence interval; LAA-GLS, left atrial appendage global longitudinal strain; LAA-VI, left atrial appendage volume index; LAVI, left atrial volume index.

**Supplementary Table S7. Net reclassification improvement and integrated discrimination improvement of LAA-VI/LAA-GLS for distinguishing Stage 1–2 from Stage 3a–3b**

| **Reference model** | **New model** | **Category-free NRI (95% CI)** | **NRI events / non-events** | **IDI (95% CI)** | ***P* (NRI / IDI)** |
| --- | --- | --- | --- | --- | --- |
| LAA-GLS | LAA-VI/LAA-GLS | 0.56 (0.27–0.97) | 0.40 / 0.16 | 0.15 (0.07–0.24) | 0.002 / <0.001 |
| LAA-VI | LAA-VI/LAA-GLS | 1.16 (0.90–1.40) | 0.43 / 0.73 | 0.39 (0.28–0.50) | <0.001 / <0.001 |
| LAVI | LAVI + LAA-VI/LAA-GLS | 1.22 (1.07–1.47) | 0.48 / 0.74 | 0.44 (0.33–0.55) | <0.001 / <0.001 |

Predicted probabilities were derived from logistic regression models with Stage 3a–3b versus Stage 1–2 as the outcome. 95% CIs and *P* values were estimated from 1,000 bootstrap resamples. Because all models were fitted and evaluated in the same cohort, these estimates are exploratory and may be optimistic. Abbreviations: IDI, integrated discrimination improvement; NRI, net reclassification improvement; other abbreviations as in Supplementary Table S6.

**Supplementary Table S8. Assessment of the proportional-odds assumption and cutpoint-specific odds ratios for LAA-VI/LAA-GLS**

| **Model** | **Brant test for LAA-VI/LAA-GLS** | **Stage ≥2 vs Stage 1, OR (95% CI)** | **Stage ≥3a vs Stage ≤2, OR (95% CI)** | **Stage 3b vs Stage ≤3a, OR (95% CI)** |
| --- | --- | --- | --- | --- |
| **Natural-log–transformed index, per 1-SD** | | | | |
| Model 1 (unadjusted) | χ² = 2.44, df = 2, *P* = 0.296 | 11.31 (5.76–22.24) | 12.47 (6.87–22.63) | 7.40 (4.45–12.31) |
| Model 2 (age, sex, BMI, LVEF, LAVI) | χ² = 2.78, df = 2, *P* = 0.249 (omnibus *P* = 0.068) | 15.00 (6.52–34.51) | 14.85 (7.21–30.61) | 7.84 (4.37–14.05) |
| Model 4 (sinus-rhythm subgroup, n = 184) | χ² = 0.03, df = 2, *P* = 0.986 | 7.60 (3.80–15.18) | 7.56 (3.98–14.37) | 8.08 (3.90–16.76) |
| **Untransformed index, per 0.1-unit** | | | | |
| Model 1 (unadjusted) | χ² = 12.45, df = 2, *P* = 0.002 | 3.46 (2.36–5.06) | 2.82 (2.18–3.64) | 1.89 (1.60–2.23) |
| Model 2 (age, sex, BMI, LVEF, LAVI) | χ² = 11.56, df = 2, *P* = 0.003 (omnibus *P* = 0.009) | 3.95 (2.49–6.27) | 2.86 (2.12–3.86) | 1.89 (1.57–2.29) |
| Model 4 (sinus-rhythm subgroup, n = 184) | χ² = 2.67, df = 2, *P* = 0.263 | 2.80 (1.90–4.12) | 2.20 (1.67–2.89) | 1.93 (1.52–2.45) |

ORs are per 1-SD increase in the natural-log–transformed index or per 0.1-unit increase in the untransformed index, as indicated, from separate cumulative binary logistic regressions at each stage threshold, with the covariates of the corresponding model; all *P* < 0.001. The Brant test could not be estimated for Model 3 because rhythm during TEE was constant (sinus rhythm) in all Stage 1–2 patients. Model numbering follows Supplementary Table S5. Abbreviations: BMI, body mass index; CI, confidence interval; LAVI, left atrial volume index; LVEF, left ventricular ejection fraction; OR, odds ratio; TEE, transesophageal echocardiography; other abbreviations as in Supplementary Table S6.

**Supplementary Table S9. Sensitivity analyses for extreme values and logarithmic transformation of LAA-VI/LAA-GLS**

| **Analysis** | **n** | **OR for higher AF stage (95% CI)** | **AIC** | **Brant test *P*** | **AUC, Stage 1–2 vs Stage 3a–3b** |
| --- | --- | --- | --- | --- | --- |
| Untransformed ratio | 267 | 2.33 (1.98–2.74) per 0.1-unit; 12.84 (7.89–20.90) per 1-SD | 558.9 | 0.002 | 0.904 |
| Excluding extreme values (> Q3 + 3 × IQR) | 265 | 2.33 (1.98–2.74) per 0.1-unit | — | 0.002 | 0.903 |
| Winsorized at 1st/99th percentiles | 267 | 2.33 (1.98–2.74) per 0.1-unit | 559.3 | 0.002 | 0.904 |
| Natural-log–transformed ratio | 267 | 9.77 (6.61–14.46) per 1-SD | 536.4 | 0.296 | 0.904 |
| Natural-log–transformed ratio, adjusted (Model 2 covariates) | 267 | 11.04 (7.17–16.98) per 1-SD | 472.8 | — | — |

ORs were derived from ordinal logistic regression with AF stage (Stage 1 < 2 < 3a < 3b) as the outcome. Model 2 covariates were age, sex, BMI, LVEF, and LAVI (AIC of the corresponding untransformed model, 490.8). AIC is not reported for the analysis excluding extreme values because it is based on a different sample. Because ROC analysis is invariant to monotonic transformation, the AUC of the log-transformed ratio is identical to that of the untransformed ratio. Abbreviations: AIC, Akaike information criterion; IQR, interquartile range; Q3, third quartile; other abbreviations as in Supplementary Tables S6 and S8.

**Supplementary Table S10. Characteristics of patients without documented AF (Stages 1–2) according to LAA-VI/LAA-GLS above or below 0.460 (mL/m²)/%**

| **Variable** | **LAA-VI/LAA-GLS > 0.460 (n = 18)** | **LAA-VI/LAA-GLS ≤ 0.460 (n = 115)** | ***P*** |
| --- | --- | --- | --- |
| AF Stage 2, n (%) | 14 (77.8) | 51 (44.3) | 0.011 |
| Age, years | 64.83 ± 8.49 | 63.85 ± 9.57 | 0.856 |
| Female, n (%) | 6 (33.3) | 42 (36.5) | 1.000 |
| Body mass index, kg/m² | 23.00 ± 2.31 | 25.02 ± 3.96 | 0.022 |
| Hypertension, n (%) | 10 (55.6) | 54 (47.0) | 0.614 |
| Diabetes mellitus, n (%) | 5 (27.8) | 16 (13.9) | 0.162 |
| Coronary heart disease, n (%) | 5 (27.8) | 10 (8.7) | 0.033 |
| CHA₂DS₂-VASc score | 2.44 ± 1.10 | 1.87 ± 1.15 | 0.052 |
| Prior stroke, TIA, or thromboembolism, n (%) | 4 (22.2) | 20 (17.4) | 0.741 |
| PFO, n (%) | 1 (5.6) | 27 (23.5) | 0.119 |
| SEC grade ≥ 1, n (%) | 12 (66.7) | 43 (37.4) | 0.023 |
| LAVI, mL/m² | 36.24 ± 4.86 | 38.06 ± 4.00 | 0.210 |
| LAD, mm | 38.00 ± 5.34 | 39.59 ± 6.30 | 0.358 |
| LVEF, % | 58.44 ± 11.79 | 57.23 ± 8.22 | 0.213 |
| LAA-VI, mL/m² | 5.50 ± 1.08 | 4.80 ± 1.28 | 0.026 |
| LAA-GLS, % | 9.21 ± 2.30 | 14.54 ± 3.45 | <0.001 |
| LAA-PEV, m/s | 0.55 ± 0.21 | 0.53 ± 0.14 | 0.919 |
| LAA-EF, % | 33.77 ± 17.32 | 33.79 ± 15.15 | 0.862 |

Values are mean ± SD or n (%). Continuous variables were compared using the Mann–Whitney U test and categorical variables using the Fisher exact test. The cutoff of 0.460 (mL/m²)/% was derived from the ROC analysis for Stage 1–2 versus Stage 3a–3b in the same cohort and is exploratory. Within Stage 2 alone, SEC grade ≥ 1 was present in 12 of 14 (85.7%) versus 43 of 51 (84.3%) patients above and below the cutoff (*P* = 1.000). Abbreviations: LAD, left atrial diameter; LAA-EF, LAA emptying fraction; LAA-PEV, LAA peak emptying velocity; PFO, patent foramen ovale; SEC, spontaneous echo contrast; TIA, transient ischemic attack; other abbreviations as in Supplementary Tables S6 and S8.

**Supplementary Table S11. Sensitivity analysis with guideline-concordant reclassification of Stage 1 patients with LAVI > 34 mL/m² as Stage 2**

| **Analysis** | **Primary staging (n = 68 / 65 / 66 / 68)** | **Reclassified staging (n = 18 / 115 / 66 / 68)** |
| --- | --- | --- |
| LAA-VI/LAA-GLS, Stage 1 | 0.33 ± 0.11 | 0.33 ± 0.09 |
| LAA-VI/LAA-GLS, Stage 2 | 0.42 ± 0.14 | 0.38 ± 0.14 |
| LAA-VI/LAA-GLS, Stage 3a | 0.59 ± 0.20 | 0.59 ± 0.20 |
| LAA-VI/LAA-GLS, Stage 3b | 0.86 ± 0.36 | 0.86 ± 0.36 |
| Ordinal logistic regression, OR per 0.1-unit (95% CI), unadjusted | 2.33 (1.98–2.74) | 2.18 (1.86–2.54) |
| Ordinal logistic regression, OR per 0.1-unit (95% CI), adjusted* | 2.39 (2.01–2.84) | 2.30 (1.94–2.74) |
| Ordinal logistic regression, OR per 1-SD of the log-transformed index (95% CI), unadjusted | 9.77 (6.61–14.46) | 7.59 (5.26–10.97) |
| Ordinal logistic regression, OR per 1-SD of the log-transformed index (95% CI), adjusted* | 11.04 (7.17–16.98) | 9.18 (6.00–14.05) |
| AUC (95% CI), Stage 1 vs Stage 2 | 0.751 (0.668–0.833) | 0.621 (0.476–0.766) |
| AUC (95% CI), Stage 1–2 vs Stage 3a–3b | 0.904 (0.868–0.941) | 0.904 (0.868–0.941) |

Values are mean ± SD (mL/m²)/% unless otherwise indicated. In the reclassified analysis, the 50 Stage 1 patients with study-measured LAVI > 34 mL/m² were reassigned to Stage 2. Analyses pooling Stages 1 and 2 (Stage 1–2 versus Stage 3a–3b and the analysis of patients without documented AF) are unaffected by this reclassification. With the log-transformed index, the proportional-odds assumption was not met after reclassification (Brant *P* = 0.013), possibly related to the small reclassified Stage 1 group (n = 18). *Adjusted for age, sex, BMI, LVEF, and LAVI. Abbreviations as in Supplementary Tables S6 and S8.

**Supplementary Table S12. Bootstrap internal validation of LAA-VI/LAA-GLS for distinguishing Stage 1–2 from Stage 3a–3b**

| **Measure** | **Apparent (original cohort)** | **Optimism** | **Optimism-corrected** |
| --- | --- | --- | --- |
| AUC | 0.904 | 0.004 | 0.900 |
| Sensitivity, % | 82.1 | 1.7 | 80.4 |
| Specificity, % | 86.5 | 1.5 | 85.0 |
| Accuracy, % | 84.3 | 1.6 | 82.7 |
| Cutoff, (mL/m²)/% | 0.460 | — | Bootstrap median 0.455 (95% interval 0.396–0.472) |
| Reselection of LAA-VI/LAA-GLS as principal index | — | — | 842/1,000 resamples (84.2%) |

Internal validation used 1,000 bootstrap resamples. In each resample, the principal index was reselected from the seven candidate LAA-SFCIs by the lowest AIC in ordinal logistic regression, and the Youden-derived cutoff was re-estimated; optimism was calculated as the mean difference between performance in the bootstrap sample and in the original cohort. In the remaining 15.8% of resamples, LAA-VI/LAA-SLE was selected. The cutoff interval is based on the resamples in which LAA-VI/LAA-GLS was selected. Abbreviations: AIC, Akaike information criterion; AUC, area under the receiver operating characteristic curve; LAA-SLE, LAA systolic longitudinal excursion; other abbreviations as in Supplementary Table S6.
